# Insecticidal Activity of Tannins from Selected Brown Macroalgae against the Cotton Leafhopper *Amrasca devastans*

**DOI:** 10.3390/plants12183188

**Published:** 2023-09-06

**Authors:** Ganeshan Petchidurai, Kitherian Sahayaraj, Laila A. Al-Shuraym, Bader Z. Albogami, Samy M. Sayed

**Affiliations:** 1 Crop Protection Research Centre (CPRC), Department of Zoology, St. Xavier’s College (Autonomous), Palayamkottai, Tirnelveli 627002, Tamil Nadu, India; durai.s.m.m@gmail.com; 2 Department of Biology, College of Science, Princess Nourah bint Abdulrahman University, P.O. Box 84428, Riyadh 11671, Saudi Arabia; 3 Department of Biology, Faculty of Arts and Sciences, Najran University, Najran P.O. Box 1988, Saudi Arabia; badrzm@hotmail.com; 4 Department of Economic Entomology and Pesticides, Faculty of Agriculture, Cairo University, Giza 12613, Egypt; samy_mahmoud@hotmail.com; 5 Department of Science and Technology, University College-Ranyah, Taif University, P.O. Box 11099, Taif 21944, Saudi Arabia

**Keywords:** brown algae, tannin, mortality, cotton leaf hopper, digestive enzymes, detoxification enzymes, total body protein

## Abstract

Seaweeds, also known as marine macroalgae, are renewable biological resources that are found worldwide and possess a wide variety of secondary metabolites, including tannins. Drifted brown seaweed (DBSW) is particularly rich in tannins and is regarded as biological trash. The cotton leaf hopper *Amrasca devastans* (Distant) has caused both quantitative and qualitative losses in cotton production. Drifted brown seaweeds (DBSWs) were used in this study to extract, qualitatively profile, and quantify the levels of total tannins, condensed tannins, hydrolyzable tannins, and phlorotannins in the seaweeds; test their insecticidal activity; and determine the mechanism of action. The largest amount of tannin extract was found in *Sargassum wightii* Greville (20.62%) using the Soxhlet method (SM). Significantly higher amounts of hydrolyzable tannins (*p* = 0.005), soluble phlorotannins (*p* = 0.005), total tannins in the SM (*p* = 0.003), and total tannins in the cold percolation method (*p* = 0.005) were recorded in *S. wightii*. However, high levels of condensed tannins (CTAs) were observed in *Turbinaria ornata* (Turner) J. Agardh (*p* = 0.004). *A. devastans* nymphs and adults were examined for oral toxicity (OT) and contact toxicity (CT) against DBSW tannin crude extract and column chromatographic fractions 1 (Rf = 0.86) and 2 (Rf = 0.88). *Stoechospermum polypodioides* (J.V. Lamouroux) J. Agardh crude tannin was highly effective against *A. devastans* using the OT method (LC_50_, 0.044%) when compared with the standard gallic acid (LC_50_, 0.044%) and tannic acid (LC50, 0.122%). Similarly, *S. wightii* fraction 2 (LC_50_, 0.007%) showed a greater insecticidal effect against *A. devastans* adults in OT than gallic acid (LC_50_, 0.034%) and tannic acid (LC_50_, 0.022%). The mechanism of action results show that *A. devastans* adults treated with crude tannin of *T. ornata* had significantly decreased amylase, protease (*p* = 0.005), and invertase (*p* = 0.003) levels when compared with the detoxification enzymes. The levels of glycosidase, lactate dehydrogenase, esterase, lipase, invertase, and acid phosphate activities (*p* = 0.005) of *S. wightii* were reduced when compared with those of the Vijayneem and chemical pesticide Monocrotophos. In adult insects treated with LC_50_ concentrations of *S. wightii* tannin fraction 1, the total body protein (9.00 µg/µL) was significantly reduced (OT, LC_50_—0.019%). The SDS-PAGE analysis results also show that *S. wightii* tannin fraction 1 (OT and CT), fraction 2 (OT), and *S. polypodioides* fraction 2 (CT) had a significant effect on the total body portion level, appearance, and disappearance of some proteins and polypeptides. This study shows that the selected brown macroalgae can be utilized for the safer management of cotton leaf hoppers.

## 1. Introduction

Benthic marine algae, often known as marine macroalgae or seaweeds, are macroscopic and multicellular. They are regarded as living and renewable resources [1]. Seaweeds come in a wide range of sizes and forms, and according to the color pigments in their cells, they can be divided into three main groups: brown algae (Ochrophyta-Phaeophyceae), green algae (Chlorophyta), and red algae (Rhodophyta) [2]. Seaweeds exhibit a wide range of secondary metabolites, including functional proteins, peptides, mycrosporine-like amino acids, alkaloids, terpenoids, carotenoids, flavonoids, saponins, and polyphenolic compounds [3].

Tannins are phenolic chemicals that are astringent, bitter, and water-soluble and are present in both angiosperms and gymnosperms [4]. According to chemical classification, tannins have three forms, namely, phlorotannins (PTs), proanthocyanidins or condensed tannins (CTAs), and hydrolyzable tannins (HTs) [3]. Both terrestrial plants and marine algae contain CTAs [5]. Seaweeds play a variety of metabolic roles, including cell wall formation, marine herbivore defense, and UV protection [6]. Additionally, seaweeds have shown insecticidal activity against many economically important pests [7,8,9,10,11,12,13]. However, this is limited to drifted seaweeds [14,15].

Phenolic compounds (gallic and tannic acids) were shown to act as insecticides [16], reduce the food consumption of fifteen species of Acridoidea, and cause death by disrupting the digestive tract’s cellular constituents [17,18]. Insects like *Orgyia leucostigma* (J E Smith) larvae [19] and *Hyphantria cunea* (Drury) [20] have developed protective mechanisms to tolerate phenolic compound ingestion. 

Drifting seaweeds represent a plant community that floats on the sea surface and moves by wind or wave action. In regions where seaweed is found, it plays a significant role in the environment. Seaweeds serve as spawning media and nurseries for different species, and they are also an effective bio-indicator of water quality [21].

The cotton leaf hopper *Amrasca devastans* (Distant) (Cicadellidae: Hemiptera) is one of the most significant, persistent, and worrying insect pests in Southern Tamil Nadu, inflicting both quantitative and qualitative losses in more than 162 economically important agricultural crops [22,23]. Insecticidal resistance to this was reported in India by various authors [24]. Hence, it is imperative to seek environmentally friendly alternative options like drifted seaweed. The drifted brown seaweed extracts, like those from *Padina pavonica* (Linnaeus) Thivy and *Sargassum wightii* Greville, demonstrated insecticidal activity against another sucking pest of cotton, namely, *Dysdercus cingulatus* (Fab.) [14]. Previously, tannins isolated from *Magonia pubescens* were shown to display larvicidal action against *Aedes aegypti* [25]. The insecticidal activity of tannins from *S. wightii*, *Stoechospermum polypodioides* (J.V. Lamouroux) J. Agardh, and *Turbinaria ornata* (Turner) J. Agardh was assessed for the first time against different life stages of *A. devastans.*

Various proteins, including enzymes, regulate the reproductive, endocrine, muscular, circulatory, respiratory, and neurological systems of insects. Enzymes are crucial for maintaining healthy physiological and biochemical metabolism, breaking down harmful substances, fending off pathogens, and maintaining physiological processes like molting, metamorphosis, slight, and diphase [26]. The actions of the digestive enzymes amylase, invertase, lipase, and protease are crucial for food and its digestion and metabolism. The brown algae species *Lobophora* sp., *Colpomenia sinuosa* (Mertens ex Roth) Derbès & Solier, *Padina gymnospora* (Kützing) Sonder, and *Dictyota* sp. inhibit the activity of digestive enzymes like catalase, superoxide dismutase, and glutathione peroxidase [27]. The literature has also revealed that tannins decrease the activity of the enzymes involved in detoxification, acetylcholine esterase, digestion, and protein synthesis in insects [28].

Even though the currently available literature on tannins provides a lengthy history, Petchidurai et al. [29] described the specifics of quantifying these different kinds of tannins from drifted seaweeds. However, there is nothing in the literature suggesting that tannins in drifted seaweeds have insecticidal properties or decrease enzyme and protein levels in insects. In this study, we investigated whether the drifting marine algae consists of phenolic compounds and whether they have insecticidal activity. Furthermore, total body protein, digestive, and detoxification enzyme levels were modulated to cause mortality and nullify the effects of phenolic compounds on cotton leaf hopper. The aim of the current study was to determine the insecticidal activity of crude tannin extracts and their column chromatographic fractions from three drifted brown seaweeds, namely, *S. wightii*, *S. polypodioides*, and *T. ornata*, against *A. devastans* using oral and contact toxicity bioassays. Furthermore, the mechanism of action was recorded to find out the total body protein level and polypeptide nature using SDS-PAGE analysis, as well as the levels of detoxification enzymes (esterase and lactase dehydrogenase) and digestive enzymes (amylase, invertase, glycosidase protease, and lipase acid phosphatase) in the whole body.

## 2. Results

### 2.1. Qualitative Profiling of Tannin Crude Extracts

All of the qualitative estimation tests revealed the presence of CTAs, HTs, soluble phlorotannins (SPTs), and total tannins (TTs) in the drifted brown seaweeds *S. wightii*, *S. polypodioides*, and *T. ornata* (Table 1).

### 2.2. Quantitative Profiling of Crude Tannin 

Considering the extraction rate, for both the Soxhlet extraction (SOE) and cold percolation (CPE) methods, the tannin extraction percentage was higher using the SOE for *S. wightti* (20.62 ± 1.20%), *S. polypodioides* (19.32 ± 5.50%), and *T. ornata* (12.16 ± 0.60%) when compared with the CPE results of *S. wightti* (17.24 ± 0.80%), *S. polypodioides* (13.84 ± 1.50%), and *T. ornata* (11.58 ± 1.10%).

**Figure 1:**
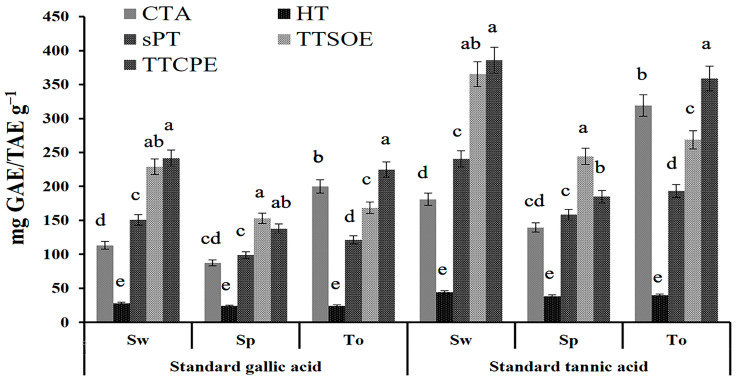
Quantitative estimation of condensed tannins (CTAs), hydrolyzable tannins (HTs), phlorotannins (PTs), soluble phlorotannins (SPTs), total tannins using the Soxhlet method (TTSOEs), and tannins using the cold percolation method (TTCPEs) (mg gallic acid (GAE) equivalents g^−1^/mg tannic acid (TAE) equivalents g^−1^) in drifted brown algae *S. wighttii* (Sw), *S*. *polypodioides* (Sp), and *T. ornata* (To). Each mean value represents the average of three replications. Bars with the same letters were not significantly different according to Tukey’s test (*p* < 0.05).

When employing the Folin–Ciocalteu method, the highest amount of CTA was recorded in *T. ornata* (F_4,25_ = 195.489, *p* = 0.005 and F_4,25_ = 193.906, *p* = 0.004 for SOE and CPE, respectively). However, greater amounts of HTs (F_4,25_ = 96.357, *p* = 0.005 and F_4,25_ = 67.106, *p* = 0.005 for SOE and CPE, respectively), SPTs (F_4,25_ = 339.489, *p* = 0.005 and F_4,25_ = 267.906, *p* = 0.005 for SOE and CPE, respectively), and TTs (F_4,25_ = 622.815, *p* = 0.005 and F_4,25_ = 715.970, *p* = 0.003 for SOE and CPE, respectively) were recorded in *S. wightii* (Figure 1).

### 2.3. Phytochemical Profiling of Seaweed Tannins

The TLC results revealed that the Rf values of gallic acid and tannic acid were 0.88 and 0.86, respectively. The Rf values in the SOE for seaweed crude extracts of *S. wightii*, *S. polypodioides*, and *T. ornata* were 0.89, 0.90, and 0.94, respectively, whereas the Rf values in the CPE for the seaweed crude extracts of *S. wightii*, *S. polypodioides*, and *T. ornata* were 0.98, 0.90, and 0.98, respectively. However, for *S. wightii*, *S. polypodioides*, and *T. ornata*, TLC analysis obtained the same Rf values for the gallic (Rf = 0.88) and tannic acid (Rf = 0.86) standards in the 50% acetone tannin fraction (Figure S1).

HPLC analyses of the tannins obtained using the SOE and CPE techniques were performed. The retention time (RT) for the typical tannins gallic acid and tannic acid were 0.671 and 0.679 min, respectively (Table 2). A single peak was observed for all tested crude (Table 2 and Figure S2) and fraction (Table 3 and Figure S3) samples with varying RTs. When comparing all three crude drifted seaweed samples and their six fractions, the fractions’ peaks were extremely similar to those of gallic and tannic acids compared with those of the crude extracts. The maximum RTs in the fractions of *S. polypodioides* and *T. ornata* were different from each other but similar to those of gallic acid. The peaks in tannic acid were substantially closer to the peaks of *T. ornata* (Rf = 0.88), *S. polypodioides*, and *S. wightii* (Table 3 and Figure S3). When compared with the crude extract, the seaweed fractions had higher levels of tannins according to the HPLC analysis. For F1, *S. wightii* had the highest quantity of tannins, followed by *S. polypodioides* and *T. ornata*. For F2, *S. polypodioides* had the highest quantity of tannins, followed by *T. ornata* and *S. wightii* (Table 3 and Figure S3).

A mass spectral identification of the peak bioactive chemicals was conducted via GC-MS utilizing the database of the National Institute of Standards and Technology (NIST). The spectra of the known bioactive components kept in the NIST collection were compared with those of the unknown bioactive components. Our tannin standard gallic acid, tannic acid crude extract, F1, and F2 contained only one compound with a different concentration, retention time, and area percentage according to the GC-MS results for all the tested brown algae (Mw 126.1100, C_6_H_6_O_3_), namely, 1,2,3-Benzenetriol. Based on area, *S. polypodioides* (80.32%) had the highest concentration of tannins, followed by the *T. ornata* (73.94%) and *S. wightii* (60.90%) crude extracts. Based on the area percentage, *S. wightii*, *S. polypodioides*, and *T. ornata* fraction 2 recorded the highest amounts of tannins (100%), while *T. ornata* F1 recorded the highest amount of tannins (65.81%), followed by *S. polypodioides* (56.68%) and *S. wightii* (18.82%) (Table 4 and Figure S4).

### 2.4. Insecticidal Activity of Crude Tannin Extract against A. devastans Adults

By means of an OT bioassay, drifted brown algal crude tannin extracts were tested against adult *A. devastans*. *S. polypodioides* significantly increased the mortality at 96 h (F_5,24_ = 59.495, *p* = 0.005) at 0.2%, followed by *T. ornata* (F_5,24_ = 30.414, *p* = 0.005) at 0.2% and *S. wightii* (F_5,24_ = 26.533, *p* = 0.005) at 0.2% when compared with the control. Tannic acid (F_5,24_ = 23.383, *p* = 0.005) and gallic acid (F_5,24_ = 22.583, *p* = 0.005) produced the statistically highest mortality. However, compared with Vijayneem, Monocrotophs caused a higher mortality ($\bar{x}$ = 84.0 ± 4.0%, F_1,4_ = 441.00, *p* = 0.005) (Table S1). According to the LC_50_ value, *S. polypodioides* (LC_50_ = 0.044%), *T. ornata* (LC_50_ = 0.058%), and *S. wightii* (LC_50_ = 0.071%) were the three best insecticidal seaweeds. The best insecticidal efficacy among the standards was demonstrated by gallic acid (LC_50_ = 0.044%), followed by tannic acid (LC_50_ = 0.122%) (Table 5).

By means of a CT bioassay, drifted brown algal crude tannin extracts were evaluated against *A. devastans* adults. At higher concentrations, *S. wightii* significantly increased the mortality (F_5,24_ = 28.565, *p* = 0.005), followed by *S. polypodioides* (F_5,24_ = 27.813, *p* = 0.005) and *T. ornata* (F_5,24_ = 40.435, *p* = 0.005). Among the standards, gallic acid caused higher mortality (F_5,24_ = 27.813, *p* = 0.005) than tannic acid. However, compared with Vijayneem, Monocrotophos caused greater mortality (F_1,4_ = 21.000, *p* = 0.005) (Table S1). *T. ornata* (LC_50_ = 0.057%) was shown to be the most effective insecticidal seaweed, followed by *S. polypodioides* (LC_50_ = 0.067%) and *S. wightii* (LC_50_ = 0.075%). Gallic acid (LC_50_ = 0.066%) showed better insecticidal activity than tannic acid (LC_50_ = 0.096%) (Table 5). 

### 2.5. Insecticidal Activity of Crude Tannin Extracts against A. devastans Nymphs 

By means of an OT bioassay, drifted brown algal crude tannin extracts were tested against third-instar *A. devastans* nymphs. At 96 h, *S. wightii* (F_5,24_ = 29.950, *p* = 0.005) and *S. polypodioides* (F_5,24_ = 25.766, *p* = 0.005) caused significantly higher mortality than *T. ornata* (F_5,24_ = 23.274, *p* = 0.005) did. Tannic acid resulted in a considerably higher mortality (F_5,24_ = 13.433, *p* = 0.005) than gallic acid (F_5,24_ = 21.890, *p* = 0.005). However, compared with Vijayneem (F_1,4_ = 112.667, *p* = 0.005), Monocrotophs caused higher mortality (F_1,4_ = 180.720, *p* = 0.005) (Table S1). Based on the LC_50_ value, *T. ornata* exhibits the best nymphicidal activity (0.070%), followed by *S. polypodioides* (0.092%) and *S. wightii* (0.106%). Gallic acid (LC_50_ = 0.083%) showed the best nymphicidal activity (LC_50_ = 0.084%) (Table 5).

By means of a CT bioassay, drifted brown algal crude tannin extracts were also tested against *A. devastans* nymphs. *S. wightii* caused significantly higher mortality at 96 h (F_5,24_ = 55.771, *p* = 0.005) (0.2%) compared with *T. ornate* and *S. polypodioides* (*p* = 0.005) (0.2%). Gallic acid ($\bar{x}$ = 80.0 ± 6.3%, F_5,24_ = 44.379, *p* = 0.005) was shown to result in a considerably higher mortality rate than tannic acid ($\bar{x}$ = 76.0 ± 7.5%, F_5,24_ = 40.217, *p* = 0.005). However, Monocrotophs ($\bar{x}$ = 80.0 ± 6.3%, F_5,24_ = 60.337, *p* = 0.005) caused higher mortality than Vijayneem ($\bar{x}$ = 56.0 ± 7.5%, F_1,4_ = 56, *p* = 0.005) (Table S1). *S. wightii* (LC_50_ = 0.071%) was found to be the most effective nymphicidal seaweed, followed by *S. polypodioides* (LC_50_ = 0.099%) and *T. ornata* (LC_50_ = 0.111%). Tannic acid (LC_50_ = 0.064%) and gallic acid (LC_50_ = 0.066%) showed the best nymphicidal efficacy (Table 5).

### 2.6. Insecticidal Activity of Column Chromatographic Fractions of Crude Tannin Extracts against A. devastans Adults

**Figure 2:**
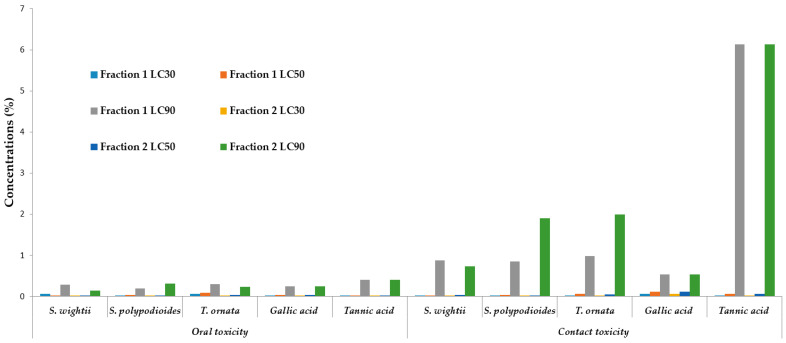
Lethal concentrations (LC%) due to tannin fractions of *S. wightii*, *S. polypodioides*, and *T. ornata* and standards (gallic acid/tannic acid) when *A. devastans* adults (male and female at random) were exposed to them during oral and contact toxicity bioassays.

The column chromatographic fractions (F1 and F2) of the tested seaweed extracts and tannin standards, namely, gallic acid and tannic acid, showed dose-dependent mortality when using OT methods. F2 of *S. wightii* caused significantly higher mortality (F_5,24_ = 34,286, *p* = 0.001), followed by *S. polypodioides* (F_5,24_ = 27.716, *p* = 0.005) and *T. ornata* (F_5,24_ = 30.439, *p* = 0.005). However, under laboratory conditions, F1 of *S. wightii* (F_5,24_ = 30.439, *p* = 0.001) and *S. polypodioides* (F_5,24_ = 13.470, *p* = 0.001) significantly killed *A. devastans* adults compared with *T. oranata* (F_5,24_ = 10.625, *p* = 0.005) (Table S2). Among the two standards, gallic acid produced noticeably higher mortality ($\bar{x}$ = 83.3.0 ± 6.1%, F_5,24_ = 17.238, *p* = 0.002) than tannic acid ($\bar{x}$ = 73.3 ± 8.4%, F_5,24_ = 15.562, *p* = 0.005) (Table S2). The results suggest that Vijayneem ($\bar{x}$ = 60.0 ± 5.2%, F_1,4_ = 135.00, *p* = 0.005) caused less mortality than Monocrotophos ($\bar{x}$ = 80.0 ± 5.2%, F_1,4_ = 240.00, *p* = 0.002) (Table S2). The results of the CT test are identical to those of the OT bioassay. When comparing F1 and F2, F2 of *S. wightii* ($\bar{x}$ = 76.7 ± 10.8%, F_5,24_ = 10.585, *p* = 0.005), *S. polypodioides* ($\bar{x}$ = 70.0 ± 6.8%, F_5,24_ = 17.626, *p* = 0.005), and *T. ornata* ($\bar{x}$ = 70.0 ± 10.0%, F_5,24_ = 8.200, *p* = 0.005) caused higher mortality than F1 of *S. wightiii* ($\bar{x}$ = 73.3 ± 8.4%, F_5,24_ = 12.529, *p* = 0.005), *S. polypodioides* ($\bar{x}$ = 66.77 ± 6.7%, F_5,24_ = 15.785, *p* = 0.005), and *T. ornata* ($\bar{x}$ = 63.3 ± 8.0%, F_5,24_ = 15.842, *p* = 0.005) (Table S2). Gallic acid caused significantly higher mortality (F_5,24_ = 8.632, *p* = 0.005), followed by tannic acid (F_5,24_ = 14.545, *p* = 0.005), Monocrotophos (F_1,4_ = 45.000, *p* = 0.005), and Vijayneem (F_1,4_ = 22.231, *p* = 0.005) (Table S2). *S. wightii* (LC_50_ = 0.007%), *S. polypodioides* (LC_50_ = 0.015%), and *T. ornata* (LC_50_ = 0.040%) were shown to be the most efficient insecticidal seaweeds against *A. devastans* adults after they were subjected to F2 during the OT bioassay, followed by F1 of *S. wightii* (LC_50_ = 0.019%), *S. polypodioides* (LC_50_ = 0.036%), and *T. ornata* (LC_50_ = 0.094%). Tannic acid (LC_50_ = 0.022%) and gallic acid (LC_50_ = 0.034%) were the two standards that demonstrated the best insecticidal performance (Table 6). Similarly, CT bioassays performed using F2 of *S. polypodioides* (LC_50_ = 0.023%), *S. wightii* (LC_50_ = 0.32%), and *T. ornata* (LC_50_ = 0.053) indicated that they were more effective insecticidal seaweeds against *A. devastans* adults than F1 of *S. wightii* (LC_50_ = 0.029%), *S. polypodioides* (LC_50_ = 0.033%), and *T. ornata* (LC_50_ =0.064%). Tannic acid (LC_50_ = 0.065%) and gallic acid (LC_50_ = 0.116%) showed the best insecticidal activities (Figure 2). Similarly, in the CT bioassays, F2 of *S. polypodioides* (LC_50_ = 0.023%) was a more effective insecticidal fraction followed by F1 of *S. wightii* (LC_50_ = 0.029%), F1 of *S. polypodioides* (LC_50_ = 0.033%), F2 of *T. ornata* (LC_50_ = 0.053), F1 of *T. ornata* (LC_50_ =0.064%), tannic acid (LC_50_ = 0.065%), gallic acid (LC_50_ = 0.116%), and F2 of *S. wightii* (LC_50_ = 0.32%) in *A. devastans* adults (Figure 2).

### 2.7. Insecticidal Activity of Column Chromatographic Fractions of Crude Tannin Extracts against A. devastans Nymphs

The tested seaweed extract fractions and tannin standards, namely, gallic acid and tannic acid, exhibited dose-dependent mortality during the OT bioassay. When compared with the control, F2 of *T. ornata* (F_5,24_ = 20.540, *p* = 0.005) caused significantly higher mortality than the other seaweed F2 fractions. No statistical difference was observed (*p* > 0.05) when comparing the mortality caused by gallic acid and tannic acid in *A. devastans* nymphs. The results suggest that the seaweed tannin extract F2 (*S. polypodioides* for CT and *T. ornata* for OT) had a greater impact on *A. devastans* nymphs than Monocrotophos or gallic acid (*p* = 0.005) (Table 6). 

The results of the CT bioassay in nymphs show that F2 of *S. polypodioides* (F_1,58_ = 4.007, *p* = 0.005), *S. wightii* (F_1,58_ = 4.007, *p* = 0.005), and *T. ornata* (F_1,58_ = 4.010, *p* = 0.005) caused greater mortality at 0.12% concentration than F1 of *S. polypodioides*, *T. ornata*, and *S. wightii* (Table 6). At a higher concentration, gallic acid caused significantly higher mortality (*p* = 0.005), followed by Monocrotophos, tannic acid, and Vijayneem (Table 6). 

**Figure 3:**
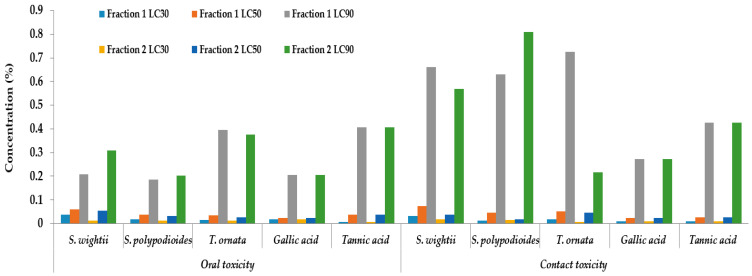
Lethal concentrations (LCs) (%) for treatments with tannin fractions of *S. wightii*, *S. polypodioides*, and *T. ornata* and standards (gallic acid and tannic acid) of *A. devastans* nymphs subjected to oral and contact toxicity bioassays.

The F2s of *T. ornata* (LC_50_ = 0.028%), *S. polypodioides* (LC_50_ = 0.032%), and *S. wightii* (LC_50_ = 0.055%) were found to be highly effective when using the OT bioassay, and these were deemed to be the best insecticidal seaweeds against *A. devastans* nymphs, compared with the F1s of *T. ornata* (LC_50_ = 0.036%), *S. polypodioides* (LC_50_ = 0.037%), and *S. wightii* (LC_50_ = 0.060%). Gallic acid (LC_50_ = 0.024%) had the strongest insecticidal effect compared with tannic acid (LC50 = 0.037%). Similarly, in the CT bioassay, the F2s of *S. polypodioides* (LC_50_ = 0.019%), *S. wightii* (LC_50_ = 0.037%), and *T. ornata* (LC_50_ = 0.045%) were shown to be better insecticidal seaweeds against *A. devastans* nymphs than the F1s of *S. polypodioides*, (LC_50_ = 0.045%), *T. ornata* (LC_50_ = 0.051%), and *S. wightii* (LC_50_ = 0.075%). Gallic acid (LC_50_ = 0.023%) and tannic acid (LC_50_ = 0.027%) had the strongest insecticidal effectiveness of the two standards (Figure 3).

### 2.8. Insecticidal Mechanism of Action of Seaweed Tannin Extracts 

*Digestive enzymes:* The results demonstrate that tannin extracts from drifting brown seaweeds strongly inhibited both the digestive and detoxifying enzymes. Tannins from *T. ornata* (0.2% concentration) considerably and significantly inhibited amylase activity ($\bar{x}$ = 0.06 ± 0.01, F_5,14_ = 104.812, *p* = 0.005). *S. wightii* (0.025% concentration) less significantly increased the amylase activity (F_5,14_ = 0.992, *p* = 0.07) (Table 7). Similar to the amylase activity, *T. ornata* (0.2% concentration) significantly decreased the protease activity ($\bar{x}$ = 20.81 ± 0.81, F_5,14_ = 271.335, *p* = 0.05). *T. ornata* (0.025% concentration) also significantly reduced the protease level ($\bar{x}$ = 122.10 ± 0.14, F_5,14_ = 271.335, *p* = 1.000) (Table 7).

At a higher concentration, *S. polypodioides* (F_6,14_ = 169.887, *p* = 0.005) and *S. wightii* (F_5,14_ = 99.862, *p* = 0.002) significantly reduced the lipase activity. *T. ornata* ($\bar{x}$ = 0.06 ± 0.01, F_5,14_ = 178.608, *p* = 0.005) and *S. wightii* ($\bar{x}$ = 0.06 ± 0.02, F_5,14_ = 165.411, *p* = 0.005) at 0.2% concentration and *S. polypodioides* at 0.025% ($\bar{x}$ = 0.34 ± 0.03, F_5,14_ = 160.743, *p* = 0.005) significantly decreased the invertase activity. *S. wightii* at 0.2% ($\bar{x}$ = 0.03 ± 0.01, F_5,14_ = 23.435, *p* = 0.005) and *S. polypodioides* at 0.025% concentration ($\bar{x}$ = 0.52 ± 0.03, F_5,14_ = 11.372, *p* = 0.005) significantly reduced the glycosidase activity. At 0.2% concentration, *S. wightii* also significantly reduced the acid phosphate activity ($\bar{x}$ = 1.08 ± 0.01, F_5,14_ = 1.031, *p* = 0.005) when compared with the standards (gallic acid/tannic acid), Vijayneem, and Monocrotophos (Table 7).

*Detoxification enzymes*: *T. ornata* (0.025% concentration) had less or equal esterase activity (F_5,14_ = 1.835, *p* = 0.005) than the control and higher activity than *S. polypodioides* (0.2% concentration) (F_5,14_ = 2.041, *p* = 0.005) (Table 8). When compared with the standards gallic acid and tannic acid, Vijayneem, Monocrotophos, and *S. polypodioides* (0.025% concentration) significantly enhanced the lactate dehydrogenase levels ($\bar{x}$ = 6.37 ± 0.07, F_5,14_ = 74.002, *p* = 0.005), and *S. wightii*, *gallic acid*, *and tannic acid* (0.2% concentration) resulted in lower esterase activity (*p* = 0.05) (Table 8).

**Figure 4:**
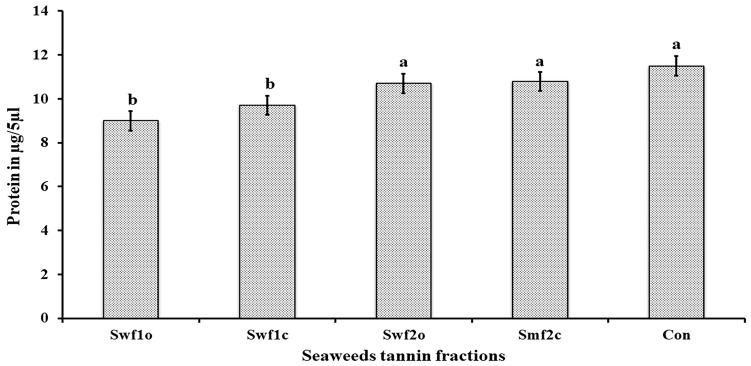
Quantification of whole-body total protein level in *A. devastans* adults treated with drifted brown seaweed tannin fractions SwF1O (*S. wightii* tannin fraction, oral toxicity LC_50_—0.019%), SwF1C (*S. wightii* tannin F1, contact toxicity LC_50_—0.029%), SwF2O (*S. wightii* tannin F2, oral toxicity LC_50_—0.007%), SpF2C (*S. polypodioides* tannin F2, contact toxicity LC_50_—0.023%), and control. Each mean value represents the average of five replications. Bars with the same letters were not significantly different according to Tukey’s test (*p* < 0.05).

*Total body macromolecular profile*: When compared with the control (11.50 µg/5 µL), *S. wightii*, F1 most significantly (OT) reduced total body protein content (9.00 µg/5 µL), followed by *S. wightii* F1 with the CT bioassay (Figure 4).

**Figure 5:**
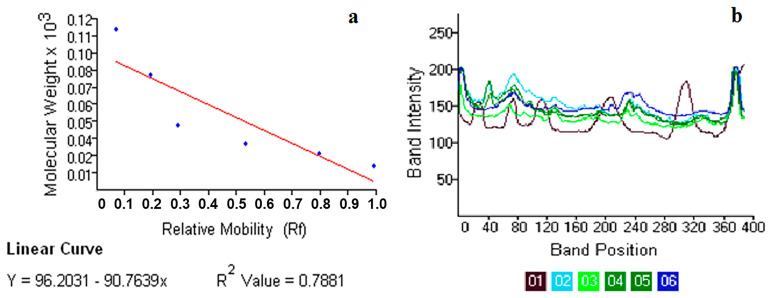
Effects of LC_50_ concentrations of seaweed tannin fractions on whole-body protein band relative mobility (**a**) and whole-body protein band intensity (**b**) of *A. devastans* adult. 1—marker, 2—control, 3—*S. wightii* tannin F1 (OT), 4—*S. wightii* tannin F1 (CT), 5—*S. wightii* tannin F2 (OT), and 6—*S. wightii* tannin F2 (CT).

*Electrophoretic analysis of whole-body total protein*: Using SDS-PAGE, 31 distinct protein bands, ranging from 8 kDa to 87 kDa, were observed in *A. devastans* adults following treatment with the seaweed tannin column chromatographic fraction (Figure S5). Nine protein bands were observed in the control *A. devastans* (8–87 kDa). *A. devastans* bioassayed according to OT and CT showed eight and nine protein bands following treatment with F1 of *S. wightii*. The Rf, area, size, raw volume, relative mobility, and band% all varied with distance (in pixels). Adult *A. devastans* treated with OT tannin fraction 1 had a high protein band percentage (Table 9, Figure S5 and Figure 5). Following treatment with *S. polypodioides* (CT) tannin F2, the band intensity was high in adult *A. devastans.* Adult *A. devastans* treated with *S. wightii* tannin F1 (OT) showed low band intensity (Figure S5 and Figure 5). In *A. devastans* treated with *S. wightii* F1, distinct protein bands of 81 kDa and 17 kDa were observed. Both *S. wightii* F1 (contact) and *S. polypodioides* F2 (CT) contained a distinct 19 kDa protein band (Table 9).

## 3. Discussion

### 3.1. Qualitative Tannin Profiling 

It was shown in the current investigation that the hydrolyzable tannin, total tannin, pholorotannin, and condensed tannin contents of drifted brown seaweeds were rich. For a total of 12 (03 × 4 = 12) tests, it was found that condensed tannins, hydrolyzable tannins, soluble pholorotannins, total tannins (SOE), and total tannins (CPE) all produced positive results, with percentages of 63.88%, 72.22%, 77.77%, 77.77%, and 63.88%, respectively. According to Melone et al. [30], the presence of HT is indicated by the 3-OH group of crude tannin extract reacting with ferric chloride to produce a black-blue color. The presence of condensed tannin is indicated by the 2-OH group of crude tannin extract reacting with ferric chloride solution to produce a greenish-grey color [31].

Although there is no difference between extraction techniques, the Soxhlet method extracted more total tannins than cold percolation techniques. This might be because the extraction process was conducted at a higher temperature, which accelerated the removal of tannin compounds (if any) from the drifting macroalgae. Other factors that affect tannin intensity from one species to another include size, age, tissue type, salinity, season, nutritional levels, herbivory intensity, light intensity, and water temperature [2]. According to Paga et al. [32], drying techniques, such as sun drying, oven drying, and freeze drying, affect the amounts of secondary metabolites, such as tannin, in *Sargassum* species. Regarding the two standards, namely, gallic acid and tannic acid, the two techniques of total tannin extraction did not significantly impact the total tannin amount purified. The majority of brown algae contained the greatest total tannin which was extracted.

### 3.2. Quantitative Tannins Profiling 

In this study, it was shown that among the four types of tannin found in drifted brown seaweeds, *S. wightii* and *S. polypodioides* were rich in total tannins, whereas *T. ornata* was rich in condensed tannins. Similarly, prior findings [29] showed that drifted *S. wightii* possesses the highest total tannins and *T. ornata* has the highest concentration of condensed tannins. Using various solvent extraction techniques, the same species’ tannin concentration varies. The rich Soxhlet extraction technique showed a high tannin level [2]. According to these findings, the yield of condensed, hydrolyzable, and phlorotannin tannin levels increased along with the increase in time and temperature. In this study, the same solvent solution was used at the same point, and neither the Soxhlet extraction method nor the cold percolation method produced the same Rf value for any seaweed crude tannin extract.

### 3.3. Tannin Analysis via HPLC

A special type of column chromatography called HPLC was used to analyze, separate, identify, and quantify the active chemicals in an extract. The crude tannins extracted using the soxhlet and cold percolation techniques and fractions were examined in this work using HPLC. A single peak was seen throughout the entire sample with varying retention times; the largest peak was found in *S. polypodioides*, *T. ornata*, and *S. wightii*. In contrast, fraction 1 of *S. wightii* showed more tannins, and Petchidurai et al. [29] found tannins using HPLC in drifted brown algae, which is similar to what was stated earlier.

### 3.4. Tannin Analysis via GC-MS

According to the GC-MS results, the crude extracts F1 and F2 of the studied brown algae contained 1,2,3-Benzenetriol, which is a synonym of pyrogallol (Catechin) 1,2,3-trihydroxybenzene, also known as pyrogallic acid. The substance has a density of 1.45 g/cm^3^, a melting temperature range of 131 °C to 134 °C, and a boiling temperature of 309 °C [33]. Gallic acid undergoes decarboxylation under high pressure and temperature to produce 1,2,3-Benzenetriol or pyrogallol. 1,2,3-Benzenetriol or pyrogallol (Catechin) was reported to be present in brown algae [34] and it showed pesticidal activity [35]. We thus tested these brown algae’s pesticidal activities against the commercially significant pest *A. devanstans*. The secretions of *A. devanstans* cause cotton leaves to become yellow, curl up, and fall off. Additionally, cotton plants develop mold as a result of their secretions. In this instance, this limits the amount of light that is able to reach the plant’s photosynthetic surfaces, lowering the yield. Nearly every year, these destructive species produce an epidemic on cotton plants. Neem oil and other natural enemies, such as ladybirds, predatory lygaeid insects, and other mantises, are frequently employed to manage this pest [36]. However, this is not feasible for large field areas. The reduction in the *A. devastans* population at the field level is at a minimum when compared with chemical control and biological techniques. We tested these seaweeds against *A. devastans* adults and nymphs under low-light conditions because marine macrobrown algae were shown to possess a variety of secondary metabolites, including tannins, condensed tannins, hydrolyzable tannins, and pholorotannins [29]. A similar dose-dependent mortality of *S. tenerrimum* crude extract and the chromatographic fraction against *D. cingulatus* nymphs was noted previously [14]. Neem extract was also noted to be effective against *A. devastans* [37].

Comparing the *S. polypodioides* crude tannin extract to the standard gallic acid and tannic acid revealed that it was much more efficient against *A. devastans* adults (Table 5). However, the OT approach showed that *T. ornata* (Table 5) was very efficient against the nymphs of *A. devastans*. TLC, HPLC, and GC-MS analyses were used to identify the presence of phenolic compounds, and the results suggested that the high polyphenol content of *T. ornata* may have been responsible for the insecticidal activity [38]. *T. ornata* also displayed anti-proliferative actions, as well as several biological characteristics (cytotoxic, antimicrobial, pesticide, etc.) [39]. The current study is the first to describe the pesticidal activity of several extracts, and their fractions demonstrated insecticidal activity. Similarly, seaweeds also showed insecticidal activity against *Spodoptera littoralis* (Boisdual) larvae [10], and the adults of *Trogoderma granarium* Everts, *Callosobruchus analis* (Fab.), *Spodoptera litura* (Fab.), *Triboliumconfusum* [40], and *S. litura* [41].

### 3.5. Insecticidal Activity of Seaweed Tannins 

*S. wightii* F2 demonstrated a stronger impact against *A. devastans* adults on the basis of the OT bioassay than the standards tannic acid and gallic acid, and in drifted brown seaweed tannin F1 (Table 6). When using a CT bioassay, F1 of *S. polypodioides* (LC_50_ = 0.019%) was more harmful to *A. devastans* nymphs than gallic acid and tannic acid (Table 8). When testing the OT and CT of *A. devastans*, the seaweed tannins F1 and F2 were found to be quite successful. Overall, the LC_50_ values of *S. wightii* F2 (Table 6) consistently exhibited a stronger impact against *A. devastans* adults in terms of the OT and CT of tannin crude extracts F1 and F2, in both nymphs and adults. *S. wightii* and *Chaetomorpha antennina* (Bory) Kützing column chromatographic fractions had a greater effect on *S. litura* larvae (*p* = 0.0001) [42] and *D. cingulatus* nymphs (86.7%, *p* = 0.004) [15], respectively. This is also supported by Sahayaraj and Mary Jeeva [14], who found that *D. cingulatus* total body protein, genomic DNA content, adult longevity, and fecundity were all decreased by *Sargassum *tenerrimum** J. Agardh extracts and chromatographic fractions. Similarly, Yu et al. [43] confirmed that the phloroglucinol, eckol, and dieckol in *Ecklonia *cava Kjellman PT** impacted mosquito DNA through H_2_O_2_-mediated DNA damage, resulting in the death of the mosquito.

Tannins in seaweeds are naturally occurring compounds that exhibit insecticidal activity through three separate mechanisms [44]. Due to their acidic nature, tannins enter an insect’s midgut or alimentary canal, where they first undergo oxidation, bind to various essential amino acids to form an insoluble protein binding complex, denature the digestive system, and ultimately cause deformities in both nymphs and adults [45]. In a second mechanism, tannin binds to lipids and carbohydrates, dramatically depleting the nutrients present, ultimately impairing the molecules’ ability to be digested and killing the insect. Third, tannins prevent insect development by causing the development of midgut lesions and releasing semiquinone and quinone free radicals as they oxidize [44].

In the CT bioassay, when an insect comes into direct contact with tannic acid, the cell membranes are disrupted; the cuticle’s chitin and wax dissolve, and the respiratory openings are blocked, which ultimately results in the death of the insect [46]. When compared with the tested brown seaweed crude and fractions, it was demonstrated using *S. polypodioides* against *A. devastans* adults (Table 6) and nymphs (Table 8) that standard tannic acid against adults (Table 6) and gallic acid against nymphs are needed in very low concentrations to cause insect mortality (Table 8). Tannic acid [17] and HTs [18] penetrate the peritrophic membrane in graminivorous species, causing damage to the midgut epithelium with the eventual development of lesions and the occurrence of tannins in the hemocoel.

### 3.6. Mechanism of Action of Insecticidal Activity of Seaweed Tannins 

*Digestive enzymes:* In this study, the activities of the digestive enzymes like amylase, protease, lipase, invertase, glycosidase, and acid phosphates, as well as the detoxification enzymes like esterase and lactate dehydrogenase, were significantly decreased when *A. devastans* adults were exposed to various concentrations of crude brown seaweed tannin extract and its fractions, gallic acid and tannic acid, and commercial pesticides [47]. The biochemistry of *S. litura* larvae is considerably impacted by bioactive chemicals derived from the seaweed *C. antennina* [42]. Based on plant species and eating behavior (e.g., omnivorous), for example, when insects consume amylase-rich plants, they have a high level of amylase activity, and the level of enzymes varies from one geographic location to another [48]. However, this is not true in our case because *A. devastans* life stages were maintained at the cotton plants, which were maintained at the Crop Protection Research Centre (CPRC), St. Xavier’s College (Autonomous), screen house. Furthermore, the insects were kept under the same lab conditions for the duration of the tests. In this study, enzyme activity was measured in adult *A. devastans* because the levels of digestive enzymes differed between males and females at various stages. Higher digestive enzyme activity was seen in the middle stages (10 and 15 days) of the female adult hemiptera insect *Apolygus lucorum* Meyer-Dur [28]. The examined drifted brown seaweed tannin crude extract revealed that with increasing tannin content, the digestive and detoxifying enzymes in *A. devastans* decreased dramatically [47].

According to Hafez and El-Naby [49], amylase is a major enzyme involved in the breakdown of carbohydrates during insect digestion. *T. ornata* crude tannin extract (0.2%) was compared with the control, gallic acid (75.75%), tannic acid (80.30%), Vijayneem (77.27%), and Monocrotophos (78.78%). The amylase activity in *A. devastans* was greatly reduced (90.91%). Similar effects on *Eurygaster integriceps* Puton were also noted in elm leaf beetles treated with *Artemisia annua* Linn. extract [50]. The findings of Sabeghi Khosroshahi et al. [51], who reported that *Phaseolus vulgaris* Linn. extract considerably decreased amylase activity in the cabbage aphid *Brevicoryne brassicae* Linn., are also supported by this information. Typically, glycosidases break down carbohydrate oligomers into monosaccharides after amylase. When compared with the control, standard gallic (68.08%) and tannic acid (38.29%), Vijayneem (36.17%), and Monocrotophos (63.82%), *S. wightii* crude tannin extract (0.2%) significantly decreased glycosidase activity (93.61%). Hemmingi and Lindroth [52] demonstrated that the glycosidase activity of *E. integriceps* adults was significantly decreased by the phenolic components of *A. annua*, as shown in *A. devastans* adults.

One of the most crucial digestive enzymes is protease. By attaching to digestive proteases in herbivorous insects, plant secondary metabolites can prevent insect protein digestion. This can lead to amino acid deficiency and proteolytic activity, which ultimately affect insect growth and development, fecundity, and survival [53]. In this study, *T. ornata* had significantly reduced protease activity (84.61%) compared with the control, which included conventional gallic (84.27%) and tannic acid (75.34%), as well as Vijayneem (59.87%) and Monocrotophos (67.48%). Leguminosae plant extract may have lowered protease activity in herbivorous pests [54,55] and a comparable outcome was also noted in that *Lathyrus sativus* Linn. and *Vicia faba* L. crude extracts decreased *Hyphantria cunea* Drury protease activity by 34.72% and 22.27%, respectively, whereas *Melia azedarach* Linn. extract inhibited *Agrotis ipsilon* Hufnagel activity (32.31%) [56].

In addition to being essential for many physiological processes underlying insect reproduction, growth, protection against pathogens, oxidative stress, and pheromone signaling, as well as providing energy during extended non-feeding periods, lipases also play key roles [49]. In this investigation, *S. polypodioides* crude tannin extract (concentration 0.2%) significantly reduced lipase activity (91.57%) compared with the control, standard gallic acid (76.19%), tannic acid (89.49%), Vijayneem (69.10%), and Monocrotophos (68.49%). Similarly, insect lipase enzyme activity was reduced by the crude extracts of azadirachtin and *A. annua* in the insect species *Cnaphalocrocis medinalis* Guenee [57]. Recent research by Liu et al. [58] demonstrated that *Zingiber officinale* Roscoe shoot extract decreased lipase activity in *Melanaphis sorghi* Schouteden. Acid phosphatases (ACPs) are critical for the completion of normal physiological processes, as well as the detoxification of toxic substances that enter insect bodies [49]. Our findings show that, in comparison with the control, gallic (67.13%) and tannic acid (79.56%), Vijayneem (35.45%), and Monocrotophos (50.70%), acid phosphatase activity was significantly reduced (88.32%) by *S. wightii* tannin crude extract at 0.2% concentration. Similarly, *Pongamia glabra* Vent. crude extract significantly decreased the acid phosphate activity of *S. litura* compared with *Christella *parasitica** (L.) H.Lev. [59]. Studies confirmed this finding by reducing the acid phosphatase activity of *Culex quinquefasciatus* Say by 34.28% when *Achyranthes aspera* Linn. extract was used [60].

One of the most significant enzymes, namely, invertase, is essential for the digestion and metabolism of carbohydrates in insects [49]. When compared with the control, standard gallic acid (82.97%) and tannic acid (57.44%), Vijayneem (72.34%), and Monocrotophos (68.08%), the invertase activity (87.23%) was significantly reduced by *T. ornata* crude tannin (0.2% concentration). Previously, *M. azedarach* and *Vinca rosea* Linn. (*Catharanthus roseus* (Linn.) (G.Don)) dramatically lowered invertase activity by 80.87% [56].

*Detoxification enzymes:* Esterases regulate juvenile hormone levels, reproduction, and nervous system function in addition to detoxifying a variety of xenobiotics, such as insecticides [61]. Esterase is a key enzyme involved in insects’ pesticide resistance processes. In this work, *S. polypodioides* crude tannin (0.2% concentration) and standard gallic acid and tannic acid (88.77%) significantly reduced the esterase activity when compared with the control, Vijayneem (84.69%), and Monocrotophos (86.73%). Azadirachtin and *M. azedarach* inhibited the esterase and acetylcholine esterase activities of *S. litura* and the hemiptera insect *Nilaparvata lugens* (Stal), respectively, at high concentrations [62]. The plant extracts *Parthenium hysterophorus* L., *Flacourtia indica*, *Chenopodiastrum murale*, *Euphorbia prostrate*, and *A. indica* were found to inhibit the acetylcholinesterase, -carboxylesterase, and β-carboxylesterase activity in larvae of the insect *Drosophila melanogaster* [63]. In addition, it was confirmed that the LC_50_ concentration of *Artemisia absinthium* crude extract reduced the acetylcholinesterase and carboxylesterase activity in larvae of *A. aegypti* [64]. Furthermore, the LC_30_ and LC_80_ of *Citrullus colocynthis* (L.) seed extract significantly inhibited esterase activity, by 45.46 and 61.16%, respectively, in *Earias vittella* [65]. The last stage of glycolysis is catalyzed by lactate dehydrogenases (LDH), which are found in almost all tissues. The lactate dehydrogenase activity was significantly reduced by *S. wightii* tannin crude extract (77.22%) compared with the standard tannins (tannic acid—73.84% and gallic acid—66.54%) and commercial pesticides (Vijayneem 61.74%, Monocrotophos 56.04%), revealing that this seaweed was not directly involved in the detoxification mechanism. *Odontopus varicornis* (Distant), which is a hemipteran bug, exhibits lactate dehydrogenase (LDH) activity inhibition throughout its whole life cycle [66]. In addition, Senthil Nathan et al. [57] demonstrated that neem seed kernel extract and *Vitex negundo* leaf extract significantly (*p* < 0.05) reduce the lactate dehydrogenase activity of *C. medinalis*.

When compared with the detoxification enzymes (esterase 88.77% and lactate dehydrogenase 77.22%), drifted seaweed significantly reduced the level of the digestive enzymes (glycosidase 93.61%, lipase 91.57%, amylase 90.99%, acid phosphate 88.32%, invertase 87.23%, and protease 84.61%) at higher concentrations. Tannins are quite beneficial for detoxifying enzymes (at low concentrations), as shown in [47]. Reduced energy metabolism and a slower rate of enzyme activity may be caused by the direct effects of plant extracts on enzyme regulation when an insect consumes the botanical extracts of *A. annua* [47]. It is clear that adult diets including seaweed tannins from plant extracts have a considerable impact on numerous enzyme activities in *A. devastans*. By detoxifying xenobiotic chemicals, detoxification enzymes are essential for plant-eating insects to continue performing their physiological functions. Gallic acid, tannins, and other secondary metabolites from *Triadica sebifera* extract have been linked to neurotoxicity, reduced insect development, and inhibition of digestive enzymes, ultimately leading to the death of *Aphis craccivora* Koch [67].

*Total body proteins:* The SDS-PAGE analysis of *A. devastans* showed that *S. wightii* tannin F1 and 2 (oral and contact toxicity) had a bigger effect than the control on the total body portion level. As a result, a few new protein bands were observed with varying intensities (SwF1 and F2-OT, SwF1 and F2-CT; Table 9). Some protein bands were shown to fade as a result of brown seaweed tannin binding with protein bodies, causing degreasing, which is why these protein bands exist [68]. A total of nine protein bands were seen in the control, whereas *A. devastans* treated with *S. wightii* tannin fraction 2 only recorded eight and nine protein bands, but *A. devastans* treated with both *S. wightii* F1 and *S. polypodioides* tannin F2 recorded eight protein bands.

## 4. Materials and Methods

### 4.1. Seaweed Collection and Preparation

Drifted seaweeds *S. wightii*, *S. polypodioides*, and *T. ornata* were gathered from Hare Island (8°46′27.9″ N, 78°11′55.7″ E), Idinthakarai (8°10′47.1″ N, 77°44′51.9″ E), and Mandapam (9°17′03.6″ N, 79°10′00.0″ E), respectively, in Tamil Nadu, India, early in the morning (5 am to 9 am) in June 2016. To remove epiphytes and sand, the collected seaweed was extensively washed in seawater, then in tap water, and finally in distilled water. Species were identified using already-existing voucher specimens and herbarium sheets available at the Crop Protection Research Centre (CPRC), St. Xavier’s College (Autonomous), and Palayamkottai [14]. The seaweeds were dried in the shade for two weeks and then processed in a home blender (Preethi XL-7, Tamil Nadu, India) to partially powder them before storing them in an airtight plastic jar (30 cm × 21 cm) for this work.

### 4.2. Extraction of Different Types of Tannins

Because it is a fairly straightforward approach that does not require any expensive equipment, CTAs, HTs, PTs, and TTs were extracted using different extraction methods, but quantified using the same method (Folin–Ciocalteu). By combining gallic acid OH and Folin–Ciocalteu to create a blue-colored molybdenum–tungsten complex, a spectrophotometer can be used to measure the extremely small amount of tannin [69]. Drifted brown seaweed tannins, including CTAs (centrifuge method), HTs (centrifuge method), SPTs (shaking method), heat-based TTs (Soxhlet technique), Rajratna, and cold-based TTs (CPE), were extracted according to the standard methodologies [70,71,72,73,74] using 70% acetone containing 0.01% L-ascorbic acid. For CT, 3 mL of a solvent mixture of acetone, water, and diethyl ether (4.7, 2.0, and 3.3) was also added for the extraction. The extraction rate was calculated by dividing the weight of the crude extract by the weight of the sample and then multiplied by 100.

### 4.3. Qualitative and Quantitative Profiling of Tannins

Condensed tannins, HTs, SPTs, and TTs, were screened using the following tests: ferric chloride [75], alcoholic ferric chloride [76], lead acetate [77], and ferrous sulfate sodium potassium tartrate [78]. Using gallic acid and tannic acid as standards, the contents of CTAs, TTs, PTs (using the Folin–Ciocalteu method), and HTs (using the potassium iodate test) were quantified using the standard methodology [71,74,79,80]. The amounts of CTAs, TTs, PTs, and HTs were expressed as mg of gallic acid g^−1^ and tannic acid g^−1^ equivalents.

### 4.4. Fractionations of Crude Tannins Using a Sephadex LH-20 Column 

The crude tannin extract was separated using a vertical glass tube column apparatus with a 1.5 cm outer diameter and a height of 15 cm. At room temperature, 2.5 g of Sephadex LH-20 (Sigma-Aldrich, Delhi, India) was swollen in 25 mL of methanol for 24 h [81]. After carefully pouring after 24 h of swelling into the column without creating any air bubbles, the column was adjusted using 30% ethanol. The column was then filled with 50 mL of 100% ethanol and 5 g of crude tannin extract. To elute the eluvants, 500 mL of 100% ethanol was used first to elute low-molecular-weight phenolic compounds, with the flow rate adjusted to 100 µL/min. The tannins were then separated from the crude extract using 300 mL of 50% acetone elution. The thin-layer chromatography fraction analyses with the same Rf value were accumulated in a 25 mL glass bottle.

### 4.5. TLC Analyses of Tannin Crude Extracts and Their Fractions

Thin-layer chromatographic analysis methods were performed as described by Helen et al. [82]. Pre-coated silica gel G 65 plates measuring 7.7 cm in height and 1.5 cm in length from Merak Specialties Private Limited in Mumbai were used for TLC analysis. Acetone, toluene, and formic acid were used as solvents (4:0.6:0.3 *v*/*v*), and 1% FeCl_3_ was used as the spraying agent. The 1% FeCl_3_ spraying resulted in the black dots being visible instantly. The Rf value was determined after the standard gallic, tannic, and crude tannin extract samples underwent TLC analysis. Ten replicates of the typical tannic and gallic acids were analyzed using TLC, and the mean values were computed. Samples were also analyzed three times using TLC, and the mean value was determined.

Rf = Distance from the source of the sample (Solute)/Distance from the source of the solvent.

Gallic acid and tannic acid standard solutions were created by dissolving 100 mg of each acid in 10 mL of HPLC-grade 99.99% methanol. The same process was also used to prepare seaweed-derived tannin samples. Then, syringe-driven filters (Hi-media) with a pore size of 0.022 µm were used to filter the standard and seaweed tannin extracts. Shimadzu Liquid Chromatography (LC-20AD with UV–Vis Detector SPD-20A, Koyoto, Japan) with a C-18 column as the stationary phase and flow rates of 1 mL/min, detection wavelengths of 280 nm, and sample injection volumes of 25 µL were used to carry out the HPLC [83].

Using a gas chromatograph interfaced with a mass spectrometer (GC-MS) (Perkin-Elmer GC System 2400, Waltham, MA, USA) equipped with an Elite-I, fused-silica capillary column (30 mm × 0.25 mm DB-5, composed of 100% dimethyl poly siloxane), drifted brown seaweed tannin crude extracts and their fractions were subjected to GC-MS analysis. An electron ionization device with an ionizing energy of 70 eV was employed for the GC-MS detection. With an injection volume of 2 µL and a split ratio of 10:1, helium gas (99.999%) was utilized as the carrier gas. The injector temperature was 100 °C, while the ion source temperature was 270 °C. With pieces ranging in size from 45 to 450 Da, mass spectra were recorded at a scan rate of 70 eV and 0.5 s. The GC was run for 36 min in total. Software called TurboMass was used to handle the mass spectra and chromatograms, and it was used to calculate the relative percent amount of each component by comparing its average peak area to the total areas [34]. The National Institute of Standards and Technology’s (NIST’s) database was used for the interpretation of the mass spectrum GC-MS data.

### 4.6. Pest Collection and Maintenance 

Adults and nymphs of *A. devastans* were collected from the cotton fields of the Tirunelveli and Tuticorin districts of Tamil Nadu, India. The insects were brought into a small screen house (5 feet high, 10 feet long, and 5 feet wide) in which cotton plants (SVPRC 5) (40 days old, 20 plants) were being maintained.

### 4.7. Determination of Insecticidal Activity via Oral Toxicity Bioassay 

The insecticidal activities against *A. devastans* adults [84] and nymphs [37] were determined using the OT method in a transparent plastic container (7.8 × 6 cm) and a glass Petri dish (15 × 3 cm), respectively, under laboratory conditions of 25 ± 2 °C and 60–70% relative humidity (RH). Adults and nymphs of *A. devastans* were selected randomly from the screen house and used for determining insecticidal activity. To test the adult and nymph oral toxicity of *A. devastans*, different concentrations of drifted crude tannin extract (0.025, 0.05, 0.1, and 0.2%) and seaweed tannin fractions (SW 86, SW 88, SM 86, SM 88, To 86, and To 88) (0.0075, 0.015, 0.03, 0.06, and 0.12%) were prepared. For the OT, fresh cotton leaves were cut into 5 × 5 cm squares. Each cotton leaf was individually submerged in the aforementioned concentrations for 5 min, after which it was allowed to dry in the shade for 5 min before being placed inside a plastic container. The cotton leaf disc in the control group was immersed in double-distilled water + Teepol. Two standard commercial insecticides, namely, Vijayneem-300 ppm (0.03%) (Madras Fertilizers Limited, Chennai, India), which is an *Azadirachata*-*indica*-based insecticide, and a chemical insecticide, namely, Monocrotophos (0.03%), were used. Adults and nymphs of *A. devastans* were released into the plastic container and Petri dish, respectively, and the mortality rate was recorded every 24 h up until they all died. Five replications were performed for the crude category and six replications for the fraction categories (each replication contained five insects, with the adults being 21 days old and the nymphs being 10 days old).

### 4.8. Determination of Insecticidal Activity via the Contact Toxicity Method

The insecticidal activity of *A. devastans* adults and nymphs [37] was determined using the CT method in a transparent plastic container (7.8 × 6 cm) and a glass Petri dish (15 × 3 cm), respectively, under laboratory conditions of 25 ± 2 °C and 60–70% relative humidity (RH). Adults and nymphs of *A. devastans* were selected randomly from the screen house and used for determining insecticidal activity. To test the adult and nymph oral toxicity of *A. devastans*, different concentrations of drifted crude tannin extract (0.025, 0.05, 0.1, and 0.2%) and seaweed tannin fractions (SW 86, SW 88, SM 86, SM 88, To 86, and To 88) (0.0075, 0.015, 0.03, 0.06, and 0.12%) were prepared. In adults and nymphs, for the contact toxicity, Whatman No. 1 filter paper was cut into pieces with dimensions of 6 and 15 cm (diameter), respectively. Each Whatman No. 1 filter paper disc was individually submerged in the aforementioned concentrations for 5 min, after which it was allowed to dry in the shade for 5 min before being placed inside a plastic container and Petri plate, respectively. The Whatman No. 1 filter paper disc in the control group was immersed in double-distilled water + Teepol. Adults and nymphs of *A. devastans* were released into the plastic container and Petri dish, respectively, and the mortality rate was recorded every 24 h up until they all died. Five replications were performed for the crude category, and six replications for the fraction categories (each replication contained five insects, each 21 days old). For the CT, fresh cotton leaf discs were then placed over the Whatman filter paper for feed. Adults and nymphs of *A. devastans* were released in a plastic container and Petri dish, respectively, and the mortality rate was noted every 24 h until they all died. Five replications in the crude category and six replication infractions were performed (with each replication containing five insects, with adults being 21 days old and nymphs being 10 days old).

### 4.9. Insecticidal Mechanism of Action

#### 4.9.1. Preparation of Enzyme Sources and Their Quantifications

Enzyme sources were prepared using the standard method. After an exposure period of 144 h, living insects were placed on normal cotton leaves and maintained under laboratory conditions for a week. Twenty healthy adult *A. devastans* insects treated with seaweed tannins were placed in a deep freezer (LG, Seoul, South Korea) for five minutes. Then, each entire body was homogenized at 4 °C with 1 mL of ice-cold phosphate buffer (1.6 g Disodium hydrogen orthophosphate + 1.1 g Sodium dihydrogen orthophosphate), in 100 mL of distilled water (pH 6.8) using a tissue homogenizer and made up to 5 mL with phosphate buffer (pH 6.8) and mixed well. The supernatant from the centrifugation of the homogenate at 5000 rpm for 15 min (Remi RM 12C, Delhi, India) was used as an enzyme source (ES). The levels of detoxification enzymes like esterase [85]; lactase dehydrogenase [86]; and digestive enzymes like amylase [87], invertase [88], glycosidase and protease [89], lipase [90], and acid phosphatase [91] were quantified using standard procedures. Five replications were performed separately for each enzyme. 

#### 4.9.2. Total Body Protein Extraction and Estimation

Twenty-five live adult *A. devastans* were treated with each concentration of *S. wightii* tannin F1 (oral toxicity LC_50_—0.007%, contact toxicity LC_50_ = 0.023%), *S. wightii* tannin F2 (oral toxicity LC_50_ = 0.019%), and *S. polypodioides* tannin F2 (contact toxicity LC_50_ = 0.023%), and control insects were homogenized in different test tubes containing Laemmli sample buffer using disposable plastic pestles. Additionally, the samples were cleared for 40 min at 4 °C at 15,000 rpm. The supernatant was gathered and then used for SDS-PAGE analysis and protein quantification. The Bradford Protein Assay was used to measure insect proteins [92]. In total, 995 µL of MilliQ water was used to prepare 5 µL samples. The samples were incubated with 1000 µL of Bradford reagent (Sigma-Aldrich, St. Louis, MO, USA) for 10 min at room temperature and in the dark. Using BSA as the protein standard, the absorbance was measured in a UV–visible spectrophotometer (JASCO V-730, Tokyo, Japan) at 595 nm. Five replications were maintained for the total body protein estimation. The amount of protein was calculated using the standard curve of BSA.

#### 4.9.3. Electrophoretic Analysis of Total Body Proteins 

In general, in accordance with Laemmli’s instructions, a 12% SDS-PAGE gel mixture was poured, run, and stained with Coomassie Blue [93]. The protein samples, which were collected in 75 µL Eppendorf tubes with sample buffer, were cooked at 100 °C for three minutes before being allowed to cool. The cooled samples were utilized as the protein samples for SDS-PAGE Gel Electrophoresis and kept in a refrigerator (LG, Seoul, South Korea) at −10 °C whenever necessary. MilliQ water (3.3 mL), 30% acrylamide (4 mL), 15 M tris pH 8.8 (2.5 mL), 10% SDS (0.1 mL), 10% APS (0.1 mL), and TEMED were combined to create 10 mL of separating gel (0.004 mL). The following ingredients were used to create a 4 mL stacking gel, MilliQ water (2.7 mL), 30% acrylamide (0.67 mL), 1 M Tris-pH 6.8, 10% SDS (0.04 mL), 10% APS (0.04 mL), and TEMED (0.004). Two glass plates were sandwiched together with spacer strips. The electrophoresis stand was used to hold the glass plate upright. The area between the glass plates was filled with 12% separating gel. About 2 cm separated the level from the notch. About 30 min were allowed for the polymerization process.

After 30 min of polymerization, stacking gel was applied over the separating gel, and a Teflon comb with seven fingers (each finger 7 mm wide) was put into the wells. The glass plates were removed from the stand that was fitted to the electrophoretic device after polymerization. The lower and top columns received an electrophoresis buffer. The Teflon comb was then slowly taken out of the gel. Using a microliter syringe, the prepared samples were added to each well in an amount of around 35 µL. A reference load of 50 µL of marker protein was placed in one well. When the sample entered the separating gel, a current of 50 volts was initially applied. Electrophoresis was then sustained at 100 volts until the marker dye reached the bottom of the separating gel. After the electrophoresis run, a stream of buffer was gently pushed between the glass plates with a spatula before the gel was transferred to a solvent-resistant plastic tray for staining. Using the gel documentation technique, the proteins in the gel were stained with Coomassie Brilliant Blue G250 for 12 h (Biotech, India) after being preserved in acetic acid (7%).

### 4.10. Statistical Analyses

All of the data (OD value, R^2^ value, mortality) were subjected to one-way analysis of variance (ANOVA), Tukey’s test was used to compare the mean values, and *p*-values of less than 0.05 were considered statistically significant using the SAS package (SPSS V16.0) [94]. The contents of condensed tannins, hydrolyzable tannins, phlorotannins, and total tannins are expressed on the basis of a standard curve of mg gallic acid equivalents g^−1^ as well as mg tannic acid equivalents g^−1^. The Abbott formula [95] was used to determine the corrected mortality. Using SPSS, the adjusted mortality data were subjected to probit analysis [94]. The regression coefficient, chi-square, and fiducidal limits (LC_30_, LC_50_, and LC_90_) were recorded.

## 5. Conclusions

In this study, total tannins, condensed tannins, hydrolyzable tannins, and phlorotannins were quantified. According to a qualitative assessment, all drifted brown algae contained TTs, SPTs, CTAs, and HTs. The amount of tannins in the drifted brown alga *S. wightii* was shown by the extraction rate. The highest amounts of TTs, SPTs, CTAs, and HTs were found in *S. wightii*. *T. ornata* had the highest concentrations of condensed tannins. Tannic acid was confirmed to be present in crude drifted brown seaweed with F1 (Rf = 0.86) and F2 (Rf = 0.88) using HPLC and GC-MS analysis. Thus, our findings support the claim that 1,2,3-benzenetriol, which is a tannic acid found in seaweeds, exerts time- and dose-dependent insecticidal activity against *A. devastans*. According to our findings, the tannins in the seaweeds *S. wightii*, *S. polypodioides*, and *T. ornata* released their insecticidal activity through a variety of mechanisms and affected adults’ normal physiological metabolism by causing numerous negative changes to several key total body proteins. The total body protein was significantly impacted, both qualitatively and quantitatively, by the dragged brown seaweed tannin components. This ingredient may be effective as a novel insecticide against this pest.

## Figures and Tables

**Table 1:** Qualitative profiling of condensed tannins (CTAs), hydrolyzable tannins (HTs), soluble phlorotannins (SPTs), and total tannins (TTs) in drifted seaweeds using ferric chloride testing (FCT), alcoholic ferric chloride testing (AFCT), lead acetate testing (LAT), and ferrous sulfate + sodium potassium tartrate testing (FSSPT) methods.

Tannins	*S. wightii*				*S. polypodioides*				*T. ornata*			
	FCT	AFCT	LAT	FSSPT	FCT	AFCT	LAT	FSSPT	FCT	AFCT	LAT	FSSPT
CTA	+	+	+++	++	++	+	+++	++	++	+	+++	++
HT	++	++	+++	++	++	++	+++	++	++	+	+++	++
SPT	++	++	+++	++	++	+	+++	+++	+++	++	+++	++
TT—Soxhlet method	++	+++	+++	++	++	+++	++	+++	++	+++	+	++
TT—cold percolation method	++	+	+++	++	++	+	+++	+	++	+	+++	++

+ slightly intense, ++ moderately intense, and +++ highly intense.

**Table 2:** HPLC analysis of crude tannin extracts of seaweeds extracted using the Soxhlet method and the cold percolation method. Retention time (RT) and area (%) are included in the table.

Seaweeds	Soxhlet Method		Cold Percolation Method	
	RT	Area	RT	Area
Gallic acid	0.671	16,505,777	-	-
Tannic acid	0.679	1,357,710	-	-
*S. wightii*	0.635	1,094,181	0.626	1,837,110
*S. polypodioides*	0.637	7,439,832	0.626	911,639
*T. ornata*	0.664	2,028,143	0.640	15,480,659

**Table 3:** HPLC analyses of tannin fractions (F1 and F2) of *S. wightii*, *S. polypodioides*, and *T. ornata*. Retention time (RT) and area (%) are included in the table.

Seaweeds	Fraction	RT	Area
Gallic acid	-	0.671	49,057,953
Tannic acid	-	0.679	62,682,156
*S. wightii*	F1	0.676	72,230,027
	F2	0.678	15,268,144
*S. polypodioides*	F1	0.678	21,539,604
	F2	0.671	56,315,135
*T. ornata*	F1	0.671	10,317,969
	F2	0.678	18,011,551

**Table 4:** GC-MS analysis of gallic acid and tannic acid; tannin crude extracts of *S. wightii*, *S. polypodioides*, and *T. ornata*; and seaweed tannin fractions *S. wightii* F1, *S. polypodioides* F1, *T. ornata* F1, *S. wightii* F2, *S. polypodioides* F2, and *T. ornata* F2. Retention time (RT) and area (%) are included in the table.

Seaweeds	RT (min)	Area (%)	RT (min)	Area (%)
Standard				
Gallic acid	5.775	17.41	-	-
Tannic acid	6.612	9.79	-	-
Tannin crude extract				
*S. wightii*	3.685	60.90	-	-
*S. polypodioides*	3.675	80.32	-	-
*T. ornata*	3.647	73.94	-	-
	Fraction 1		Fraction 2	
*S. wightii*	5.862	18.82	3.637	100.00
*S. polypodioides*	5.772	56.68	3.638	100.00
*T. ornata*	5.752	65.81	3.704	100.00

1,2,3-Benzenetriol (C_6_H_3_(OH)_3_; Mw—126.1100).

**Table 5:** Insecticidal activity (oral and contact toxicity bioassays) of crude tannin extracts of *S. wightii*, *S. polypodioides*, and *T. ornata* and standards (gallic acid and tannic acid) on nymphs and adult (male/female at random) of *A. devastans.* Lethal concentration for 50% mortality (LC_50_), 95% confidence interval (CI), slope, chi-squared values, and significance values at 5% level when subjected to Tukey’s test. Χ^2^—chi-squared value, *p*—probability, ns—not significant.

Seaweeds Extracts	Adult					Nymph				
	LC_50_	CI	Χ^2^	Slope	*p*	LC_50_	CI	Χ^2^	Slope	*p*
	Oral Toxicity									
*S.wightii*	0.071	0.024–0.316	3.820	1.35 ± 0.84	0.051	0.106	0.049–0.287	37.976	2.91 ± 0.14	0.005
*S. polypodioides*	0.044	0.023–0.432	0.022	1.17 ± 0.19	0.881 ns	0.092	0.020–0.318	68.751	2.72 ± 0.17	0.005
*T. ornata*	0.058	0.028–0.722	0.493	1.26 ± 0.10	0.481 ns	0.070	0.042–0.160	74.502	1.59 ± 0.62	0.005
Gallic acid	0.044	0.014–0.232	2.192	1.16 ± 0.58	0.139 ns	0.083	0.021–0.397	81.371	1.77 ± 0.13	0.005
Tannic acid	0.122	0.067–1.000	0.035	1.82 ± 0.13	0.130 ns	0.084	0.024–0.273	48.448	1.86 ± 0.50	0.005
	Contact toxicity									
*S. wightii*	0.075	0.021–0.344	46.499	1.97 ± 0.22	0.000	0.071	0.036–0.355	2.060	2.86 ± 0.89	0.151 ns
*S. polypodioides*	0.067	0.006–0.325	58.115	1.86 ± 0.16	0.000	0.099	0.040–0.736	6.047	2.14 ± 0.52	0.014 ns
*T. ornata*	0.057	0.037–0.284	85.368	1.72 ± 0.42	0.000	0.111	0.040–0.296	5.529	2.41 ± 0.54	0.019 ns
Gallic acid	0.066	0.001–0.269	69.111	1.80 ± 0.18	0.000	0.066	0.026–0.103	0.616	1.48 ± 0.33	0.414 ns
Tannic acid	0.096	0.015–0.350	59.423	2.64 ± 0.17	0.000	0.064	0.026–0.097	1.559	3.02 ± 0.71	0.212 ns

**Table 6:** Impacts of tannin fractions (F1 and F2) of *S. wightii*, *S. polypodioides*, and *T. ornata*; standards gallic acid/tannic acid (0.0075, 0.015, 0.03, 0.06 and 0.12%); and commercial insecticides (Vijayneem/Monocrotophos—0.03%) on *A. devastans* nymphal mortality (%) during oral and contact toxicity bioassays (n = 30, $\bar{x}$ ± SE).

Seaweed Extract /Tannin Standards	Concentrations	Oral Toxicity—Nymph		Contact Toxicity—Nymph	
		F1	F2	F1	F2
*S. wightii*	0.0075	6.7 ± 0.4 ^eA^	6.7 ± 0.4 ^eA^	23.3 ± 9.5 ^eA^	20.0 ± 7.3 ^deB^
	0.015	16.7 ± 6.1 ^dB^	36.7 ± 8.0 ^dA^	36.7 ± 9.5 ^dA^	26.7 ± 6.7 ^dB^
	0.03	33.3 ± 11.1 ^cB^	50.0 ± 8.6 ^cA^	40.0 ± 8.9 ^cA^	40.0 ± 10.3 ^cA^
	0.06	43.3 ± 3.3 ^bB^	56.7 ± 9.5 ^bA^	50.0 ± 1.2 ^bB^	63.3 ± 9.5 ^bA^
	0.12	60.0 ± 5.2 ^aB^	63.3 ± 2.0 ^aA^	63.3 ± 6.1 ^aB^	80.0 ± 7.3 ^aA^
*S. polypodioides*	0.0075	16.7 ± 6.1 ^eA^	10.0 ± 4.4 ^eB^	13.3 ± 4.2 ^eA^	06.7 ± 4.2 ^eB^
	0.015	33.3 ± 12.3 ^dB^	40.0 ± 7.3 ^dA^	43.3 ± 9.5 ^cdA^	30.0 ± 8.5 ^dB^
	0.03	56.7 ± 10.8 ^cA^	50.0 ± 8.5 ^bcB^	46.7 ± 9.9 ^cA^	43.3 ± 2.0 ^cB^
	0.06	63.3 ± 8.0 ^abA^	53.3 ± 6.1 ^bB^	70.0 ± 1.0 ^bA^	70.0 ± 8.5 ^bA^
	0.12	63.3 ± 3.3 ^aA^	63.3 ± 1.1 ^aA^	80.0 ± 5.9 ^aB^	83.3 ± 6.1 ^aA^
*T. ornata*	0.0075	13.3 ± 4.2 ^eB^	26.7 ± 6.7 ^eA^	20.0 ± 5.1 ^eA^	16.7 ± 6.1 ^eB^
	0.015	30.0 ± 4.4 ^dB^	50.0 ± 6.8 ^dA^	36.7 ± 4.0 ^dA^	30.0 ± 4.5 ^dB^
	0.03	43.3 ± 6.1 ^cB^	60.0 ± 1.5 ^cA^	46.7 ± 6.6 ^cB^	50.0 ± 2.0 ^cA^
	0.06	50.0 ± 10.0 ^bB^	73.3 ± 4.2 ^bA^	63.3 ± 9.5 ^bA^	60.0 ± 8.9 ^bB^
	0.12	66.7 ± 6.7 ^aB^	83.3 ± 5.4 ^aA^	73.3 ± 1.1 ^aB^	76.7 ± 8.0 ^aA^
Gallic acid	0.0075	23.3 ± 8.0 ^e^		20.0 ± 8.9 ^e^	
	0.015	46.6 ± 6.7 ^d^		33.3 ± 6.7 ^d^	
	0.03	56.6 ± 6.7 ^c^		46.7 ± 6.7 ^c^	
	0.06	70.0 ± 10.0 ^b^		70.0 ± 8.6 ^b^	
	0.12	80.0 ± 7.3 ^a^		83.3 ± 8.0 ^a^	
Tannic acid	0.0075	20.0 ± 5.1 ^e^		30.0 ± 4.5 ^e^	
	0.015	50.0 ± 10.0 ^cd^		36.7 ± 6.1 ^d^	
	0.03	50.0 ± 12.4 ^c^		56.7 ± 9.5 ^c^	
	0.06	63.3 ± 9.5 ^b^		73.3 ± 1.1 ^ab^	
	0.12	76.7 ± 8.0 ^a^		73.3 ± 9.9 ^a^	
Vijayneem	0.03	63.3 ± 13.1 ^b^		66.7 ± 2.3 ^b^	
Monocrotophos	0.03	70.0 ± 16.9 ^a^		76.7 ± 6.1 ^a^	

Same letter in the same column—no significant differences and different letter—significant differences at 0.05% level with Tukey’s test. The uppercase letters indicate the comparison between F1 and F2 of oral and contact toxicity bioassays of adults and nymphs separately.

**Table 7:** Whole-insect-body digestive enzyme levels in *A. devastans* adults treated with different concentrations (0.025, 0.05, 0.1, 0.2) of tannins from different drifted brown algae *S. wightii*, *S. polypodioides*, and *T. ornata*; gallic acid and tannic acid; and commercial insecticides Monocrotophos and Vijayneem (0.03%) (n = 25, $\bar{x}$ ± SE) for oral toxicity bioassay.

Seaweed Name	Concentrations	Amylase	Protease	Lipase	Invertase	Glycosidase	Acid Phosphates
*S. wightii*	0.025	0.83 ± 0.11 ^a^	80.15 ± 0.10 ^ab^	6.41 ± 0.07 ^b^	0.26 ± 0.01 ^b^	0.50 ± 0.01 ^b^	7.02 ± 0.05 ^b^
	0.05	0.41 ± 0.01 ^c^	60.21 ± 0.02 ^bc^	5.30 ± 0.13 ^c^	0.15 ± 0.05 ^c^	0.22 ± 0.02 ^c^	5.03 ± 0.10 ^c^
	0.1	0.34 ± 0.02 ^d^	43.12 ± 0.08 ^cd^	3.11 ± 0.05 ^d^	0.12 ± 0.01 ^cd^	0.05 ± 0.01 ^d^	2.35 ± 0.02 ^d^
	0.2	0.23 ± 0.01 ^e^	35.62 ± 0.03 ^de^	1.63 ± 0.01 ^de^	0.06 ± 0.02 ^e^	0.03 ± 0.01 ^de^	1.08 ± 0.01 ^e^
*S. polypodioides*	0.025	0.41 ± 0.01-^b^	81.08 ± 0.01 ^b^	5.61 ± 0.07 ^b^	0.34 ± 0.03 ^b^	0.52 ± 0.03 ^b^	7.19 ± 0.04 ^b^
	0.05	0.23 ± 0.02 ^c^	75.35 ± 0.04 ^c^	3.38 ± 0.03 ^c^	0.24 ± 0.05 ^b^	0.31 ± 0.01 ^c^	5.73 ± 0.03 ^c^
	0.1	0.15 ± 0.01 ^d^	52.96 ± 0.01 ^d^	1.15 ± 0.01 ^d^	0.18 ± 0.02 ^d^	0.23 ± 0.02 ^d^	3.72 ± 0.03 ^d^
	0.2	0.13 ± 0.01 ^e^	27.97 ± 0.02 ^e^	0.69 ± 0.01 ^de^	0.13 ± 0.01 ^de^	0.14 ± 0.01 ^e^	1.61 ± 0.01 ^e^
*T. ornata*	0.025	0.57 ± 0.02 ^b^	122.10 ± 0.14 ^b^	5.17 ± 0.15 ^b^	0.22 ± 0.02 ^b^	0.32 ± 0.05 ^b^	8.07 ± 0.11 ^b^
	0.05	0.31 ± 0.04 ^c^	83.35 ± 0.01 ^c^	3.93 ± 0.03 ^c^	0.18 ± 0.01 ^c^	0.21 ± 0.03 ^c^	6.74 ± 0.06 ^c^
	0.1	0.16 ± 0.01 ^d^	33.17 ± 0.02 ^d^	2.96 ± 0.02 ^d^	0.11 ± 0.03 ^d^	0.14 ± 0.01 ^d^	5.93 ± 0.02 ^d^
	0.2	0.06 ± 0.01 ^de^	20.81 ± 0.02 ^e^	2.84 ± 0.02 ^de^	0.06 ± 0.01 ^de^	0.10 ± 0.01 ^de^	3.15 ± 0.05 ^e^
Galic acid	0.025	0.30 ± 0.01 ^b^	93.14 ± 0.02 ^b^	4.96 ± 0.02 ^b^	0.22 ± 0.01 ^b^	0.37 ± 0.02 ^b^	7.75 ± 0.05 ^b^
	0.05	0.26 ± 0.01 ^c^	64.01 ± 0.01 ^c^	3.75 ± 0.01 ^c^	0.18 ± 0.03 ^c^	0.32 ± 0.01 ^bc^	6.26 ± 0.03 ^c^
	0.1	0.16 ± 0.01 ^d^	44.34 ± 0.04 ^d^	1.87 ± 0.03 ^d^	0.14 ± 0.05 ^cd^	0.28 ± 0.03 ^d^	4.65 ± 0.01 ^d^
	0.2	0.16 ± 0.01 ^de^	21.26 ± 0.02 ^e^	1.95 ± 0.00 ^de^	0.08 ± 0.01 ^e^	0.15 ± 0.01 ^e^	3.04 ± 0.05 ^e^
Tannic acid	0.025	0.50 ± 0.02 ^b^	92.23 ± 0.05 ^b^	3.79 ± 0.09 ^b^	0.32 ± 0.05 ^b^	0.49 ± 0.01 ^b^	7.42 ± 0.30 ^b^
	0.05	0.33 ± 0.03 ^c^	74.72 ± 0.03 ^c^	2.43 ± 0.10 ^c^	0.30 ± 0.02 ^c^	0.43 ± 0.04 ^c^	5.56 ± 0.02 ^c^
	0.1	0.20 ± 0.02 ^d^	51.37 ± 0.01 ^d^	1.95 ± 0.03 ^d^	0.21 ± 0.03 ^d^	0.38 ± 0.01 ^d^	3.87 ± 0.02 ^d^
	0.2	0.13 ± 0.01 ^e^	33.34 ± 0.01 ^e^	0.86 ± 0.01 ^e^	0.20 ± 0.05 ^e^	0.29 ± 0.02 ^e^	1.89 ± 0.02 ^e^
Vijayneem	0.03	0.15 ± 0.01 ^c^	54.27 ± 0.07 ^b^	2.53 ± 0.03 ^bc^	0.13 ± 0.01 ^bc^	0.30 ± 0.01 ^b^	5.97 ± 0.02 ^b^
Monocrotophos	0.03	0.14 ± 0.02 ^bc^	43.97 ± 0.01 ^c^	2.58 ± 0.08 ^b^	0.15 ± 0.02 ^b^	0.17 ± 0.03 ^c^	4.56 ± 0.02 ^bc^
Control	0.00	0.66 ± 0.04 ^a^	135.24 ± 0.12 ^a^	8.19 ± 0.05 ^a^	0.47 ± 0.03 ^a^	0.47 ± 0.10 ^a^	9.25 ± 1.40 ^a^

Amylase, protease, lipase (µg/mg), invertase (mg glucose/mg protein/h), glycosidase (µL/min/mg), and acid phosphate (µmoles of phenol released/min/mg protein) levels, expressed in standard units. Same letter in the same column—no significant differences and different letters—significant differences at 0.05% level tested using a post-ANOVA Tukey’s test.

**Table 8:** Whole-body detoxification enzyme levels of *A. devastans* adults treated with different concentrations (0.025, 0.05, 0.1, 0.2) of crude tannin extracts of *S. wightii*, *S. polypodioides*, and *T. ornata;* standards (gallic acid, tannic acid); commercial insecticides (Monocrotophos and Vijayneem-0.03%); and untreated control during oral toxicity bioassay.

Seaweeds Name	Concentrations	Esterase	Lactate Dehydrogenase
*S. wightii*	0.025	0.15 ± 0.02 ^a^	3.51 ± 0.03 ^a^
	0.05	0.14 ± 0.01 ^ab^	2.44 ± 0.02 ^b^
	0.1	0.13 ± 0.03 ^c^	2.46 ± 0.02 ^bc^
	0.2	0.13 ± 0.02 ^cd^	1.28 ± 0.02 ^d^
*S. polypodioides*	0.025	0.16 ± 0.02 ^a^	6.37 ± 0.07 ^a^
	0.05	0.14 ± 0.01 ^ab^	5.66 ± 0.02 ^b^
	0.1	0.12 ± 0.01 ^c^	4.84 ± 0.01 ^c^
	0.2	0.11 ± 0.02 ^cd^	4.26 ± 0.05 ^cd^
*T. ornata*	0.025	0.94 ± 0.21 ^a^	4.91 ± 0.01 ^a^
	0.05	0.93 ± 0.11 ^ab^	4.44 ± 0.05 ^ab^
	0.1	0.83 ± 0.10 ^c^	4.28 ± 0.03 ^c^
	0.2	0.73 ± 0.14 ^d^	3.52 ± 0.05 ^d^
Galic acid	0.025	0.15 ± 0.05 ^a^	2.50 ± 0.02 ^a^
	0.05	0.12 ± 0.01 ^ab^	1.73 ± 0.04 ^b^
	0.1	0.12 ± 0.01 ^bc^	1.56 ± 0.01 ^c^
	0.2	0.11 ± 0.02 ^cd^	1.88 ± 0.01 ^cd^
Tannic acid	0.025	0.15 ± 0.02 ^a^	4.64 ± 0.03 ^a^
	0.05	0.14 ± 0.01 ^ab^	3.17 ± 0.05 ^b^
	0.1	0.12 ± 0.02 ^bc^	2.79 ± 0.04 ^c^
	0.2	0.11 ± 0.05 ^cd^	1.47 ± 0.02 ^d^
Vijayneem	0.03	0.15 ± 0.03 ^bc^	2.15 ± 0.04 ^bc^
Monocrotophos	0.03	0.13 ± 0.01 ^b^	2.47 ± 0.03 ^b^
Control	0.00	0.98 ± 0.02 ^a^	5.62 ± 0.06 ^a^

Esterase level expressed in µM product/min/mg protein and lactate dehydrogenase level expressed in µmoles protein/mg). Same letter—no significant differences and different lettersignificant differences at 0.05% level tested using post-ANOVA Tukey’s test.

**Table 9:** *A. devastans* adult whole-body protein molecular weight determination with distance (pixels), Rf, area estimated size, raw volume, and band (%) treated with seaweed algal tannin fractions (F1 and F2).

Marker									
Band/Line	1	2	3	4	5	6	7	8	9
Distance (pixels)	27	74	113	206	309	385	-	-	-
Rf	0.070	0.191	0.292	0.532	0.798	0.995	-	-	-
Area	867	765	1173	1938	1734	1122	-	-	-
Size	113	81	46	33	26	17	-	-	-
Raw volume	147,415	133,002	195,130	308,315	268,945	186,601	-	-	-
Band %	11.89	10.73	15.74	24.88	21.7	15.06	-	-	-
Control									
Distance (pixels)	41	73	84	130	190	232	243	322	377
Rf	0.106	0.189	0.217	0.336	0.491	0.599	0.628	0.832	0.974
Area	1007	848	795	795	901	636	530	1484	1060
Size	87	79	77	66	52	42	39	21	8
Raw volume	183,762	164,300	144,290	140,081	156,148	120,430	98,952	242,149	206,103
Band %	12.62	11.28	9.91	9.62	10.72	8.27	6.8	16.63	14.15
*S. wightii* tannin F1 (OT)									
Distance (pixels)	67	89	128	188	228	241	336	376	-
Rf	0.173	0.23	0.331	0.486	0.589	0.623	0.868	0.972	-
Area	583	477	954	689	689	689	1484	1007	-
Size	81	75	66	52	43	40	17	8	-
Raw volume	105,928	84,023	161,879	114,650	119,240	117,036	232,630	191,000	-
Band %	9.4	7.46	14.37	10.18	10.59	10.39	20.65	16.96	-
*S. wightii* tannin F1 (CT)									
Distance (pixels)	41	75	88	130	190	232	242	330	374
Rf	0.106	0.194	0.227	0.336	0.491	0.599	0.625	0.853	0.966
Area	1311	1026	798	912	1026	570	684	1482	741
Size	87	79	76	66	52	42	39	19	9
Raw volume	238,608	197,107	145,182	160,235	175,741	107,944	123,505	237,000	150,656
Band %	15.53	12.83	9.45	10.43	11.44	7.03	8.04	15.43	9.81
*S. wightii* tannin F2 (OT)									
Distance (pixels)	43	73	92	129	192	232	244	377	-
Rf	0.111	0.189	0.238	0.333	0.496	0.599	0.63	0.974	-
Area	784	1120	896	1064	616	672	728	784	-
Size	86	79	75	66	51	42	39	8	-
Raw volume	142,491	204,140	157,857	180,277	108,466	123,369	129,120	155,170	-
Band %	11.87	17	13.15	15.01	9.03	10.27	10.75	12.92	-
*S. wightii* tannin F2 (CT)									
Distance (pixels)	75	88	131	186	232	245	329	377	-
Rf	0.194	0.227	0.339	0.481	0.599	0.633	0.85	0.974	-
Area	1218	638	1218	1044	1102	1160	928	1218	-
Size	79	76	65	53	42	39	19	8	-
Raw volume	230,090	118,225	213,365	181,762	203,929	204,665	155,950	233,939	-
Band %	14.92	7.67	13.84	11.79	13.23	13.27	10.11	15.17	-

## Data Availability

The data presented in this study are available on request from the corresponding author.

## Supplementary Materials

The following supporting information can be downloaded from https://www.mdpi.com/article/10.3390/plants12183188/s1: Table S1. Insecticidal activity of crude tannin extracts of *S. wightii*, *S. polypodioides*, and *T. ornata* (0.025, 0.05, 0.1, and 0.2%); standards (gallic acid and tannic acid) (0.025, 0.05, 0.1 and 0.2%); and commercial insecticides (Vijayneem and Monocrotophos-0.03%) on *A. devastans* nymphs and adults (male/female at random) mortality (%) after 96 h exposure on the basis of oral and contact toxicity bioassays (n = 25, $\bar{x}$ ± SE). Table S2. Impact of tannin fractions (F1 Rf—0.86 and F2 Rf—0.88) of *S. wightii*, *S. polypodioides*, and *T. ornata;* standards (gallic acid/tannic acid) (0.0075, 0.015, 0.03, 0.06 and 0.12%); and commercial insecticides (Vijayneem/Monocrotophos-0.03%) on *A. devastans* adult (male/female at random) mortality (%) after 96 h exposure during oral and contact toxicity bioassays (n = 30, $\bar{x}$ ± SE). Figure S1. Photograph showing the TLC analysis of the standards (a) gallic acid and (b) tannic acid, along with (c) *S. wightii* F1, (d) *S. polypodioides* F1, (e) *T. ornata* F1, (f) *S. wightii* F2, (g) *S. polypodioides* F2, and (h) *T. ornata* F2. Tannic and gallic acids were processed in TLC 10 times, and samples were processed three times in TLC. Figure S2. HPLC analysis of standards gallic acid (a) and tannic acid (b), and crude tannins extracted from *S. wightii* (c), *S. polypodioides* (d), and *T. ornata* (e) using the Soxhlet method and *S. wightii* (f), *S. polypodioides* (g), and *T. ornata* (h) using the cold percolation method. Figure S3. HPLC analysis of gallic acid (a) and tannic acid (b), along with brown algae *S. wightii* F1 (c), *S. wightii* F2 (d), *S. polypodioides* F1 (e), *S. polypodioides* F2 (f), *T. ornata* F1 (g), and *T. ornata* F2 (h). Figure S4. GC-MS analysis of gallic acid (a) and tannic acid (b), and drifted brown seaweed tannin crude extracts *S. wightii* (c), *S. polypodioides* (d), and *T. ornata* (e), along with *S. wightii* F1 (f), *S. polypodioides* F1 (g), *T. ornata* F1 (h), *S. wightii* F2 (i), *S. polypodioides* F2 (j), and *T. ornata* F2 (k). Figure S5. Effects of LC_50_ concentrations of seaweed tannin fractions on whole-body protein SDS-PAGE of *A. devastans* adults. C—control, SWF1O—*S. wightii* tannin F1 (oral toxicity), SWF2C—*S. wightii* tannin F1 (contact toxicity), SWF2O—*S. wightii* tannin F2 (oral toxicity), SWF2C—*S. wightii* tannin F2 (contact toxicity), and M—marker.
