# Supplementary material for: Insecticidal Activity of Tannins from Selected Brown Macroalgae against the Cotton Leafhopper Amrasca devastans
Source: Plants (Basel). 2023 Sep 6;12(18):3188. doi: 10.3390/plants12183188 (PMC10537709; doi:10.3390/plants12183188)
Supplement: Supplementary file 1 [file plants-12-03188-s001.zip › plants-2487415-supplementary.pdf]

plants

Supplementary Materials

Table S1. Insecticidal activity of crude tannin extracts of *S. wightii*, *S. polypodioides* and *T. ornata* (0.025, 0.05, 0.1 and 0.2%), standards (gallic acid and tannic acid) (0.025, 0.05, 0.1 and 0.2%) and commercial insecticides (Vijayneem and Monocrotophos-0.03%) on *A. devastans* nymphs and adults (male/female at random) mortality (%) after 96 h exposure on the basis of oral and contact toxicity bioassays (n = 25, $\bar{x}$ ± SE).

Seaweeds	Concentrations (%)	Adults		Nymphs	
		Oral toxicity	Contact toxicity	Oral toxicity	Contact toxicity
S. wightii	0.025	28.0±4.9^{cB}	32.0±8.0^{cdA}	16.0±4.0^{dB}	20.0±6.3^{dA}
	0.05	44.0±7.5^{bA}	40.0±8.9^{cB}	32.0±10.2^{cB}	44.0±4.0^{cA}
	0.1	64.0±7.5^{aA}	60.0±8.9^{bB}	56.0±7.5^{bA}	56.0±4.0^{bA}
	0.2	64.0±7.5^{aB}	88.0±4.9^{aA}	76.0±4.0^{aAB}	80.0±6.3^{Aa}
S. polypodioides	0.025	36.0±7.5^{dB}	40.0±10.9^{cdA}	24.0±9.0^{dA}	16.0±4.9^{daB}
	0.05	56.0±4.0^{cA}	44.0±7.4^{cB}	52.0±10.2^{bcA}	40.0±6.3^{cB}
	0.1	76.0±7.5^{bA}	68.0±4.9^{bB}	56.0±4.0^{bA}	56.0±9.9^{bA}
	0.2	88.0±4.9^{aA}	84.0±7.5^{aAB}	76.0±4.0^{aA}	60.0±14.2^{aB}
T. ornata	0.025	28.0±8.0^{bD}	40.0±0.1^{dA}	36.0±11.7^{dA}	12.0±4.9^{dB}
	0.05	44.0±7.5^{bC}	60.0±0.1^{cA}	56.0±7.5^{cA}	36.0±7.5^{cB}
	0.1	68.0±4.9^{bB}	72.0±10.2^{abA}	60.0±1.2^{bA}	52.0±4.9^{bB}
	0.2	80.0±9.0^{aA}	80.0±6.3^{aA}	72.0±4.0^{aA}	60.0±6.3^{aB}
**Gallic acid**	0.025	36.0±4.0^{aD}	36.0±3.9^{dA}	32.0±1.8^{dA}	20.0±6.3^{dB}
	0.05	56.0±9.8^{cA}	54.0±7.5^{cAB}	56.0±7.5^{cA}	44.0±4.0^{cB}
	0.1	68.0±4.9^{abA}	64.0±7.5^{bB}	72.0±8.0^{abA}	64.0±7.4^{bB}
	0.2	72.0±2.0^{aB}	84.0±11.7^{aA}	76.0±9.8^{aB}	80.0±6.3^{aA}
**Tannic acid**	0.025	36.0±7.5^{cdA}	32.0±12.0^{dB}	28.0±4.9^{dA}	28.0±4.9^{dA}
	0.05	44.0±7.5^{cB}	48.0±4.9^{cA}	40.0±2.6^{cB}	48.0±4.9^{cA}
	0.1	60.0±9.0^{bA}	56.0±7.5^{bB}	64.0±3.2^{bA}	56.0±7.5^{bB}
	0.2	76.0±7.5^{aA}	72.0±3.8^{aB}	80.0±0.9^{aA}	76.0±7.5^{bAB}
**Vijayneem**	0.03	60.0±6.3^{aA}	52.0±4.9^{aB}	52.0±4.9^{aA}	56.0±7.5^{aA}
**Monocrotophos**	0.03	84.0±4.0^{aA}	72.0±5.7^{bB}	80.0±8.9^{aA}	80.0±6.3^{aA}

The same lowercase letters in a column for each plants and standards separately indicate no significant difference in the Tukey test ($P < 0.05$). The same uppercase letters indicate no significant difference in the Tukey test ($P < 0.05$) between oral and contact toxicity bioassays of adults and nymphs separately.

Table S2. Impact of tannin fractions (F1 Rf—0.86 and F2 Rf—0.88) of *S. wightii*, *S. polypodioides* and *T. ornata;* standards (gallic acid/tannic acid) (0.0075, 0.015, 0.03, 0.06 and 0.12%) and commercial insecticides (Vijayneem/Monocrotophos-0.03%) on *A. devastans* adult (male/female at random) mortality (%) after 96 h exposure during oral and contact toxicity bioassays (n = 30, $\bar{x}$ ± SE).

Seaweeds extract /tannin standards	Concentrations	Oral toxicity—adult		Contact toxicity—adult	
		Fraction 1	Fraction 2	Fraction 1	Fraction 2
S. wightii	0.0075	46.7±4.1^{dA}	30.0±8.6^{eB}	33.3±12.3^{eB}	40.0±10.3^{deA}
	0.015	70.0±6.8^{cA}	46.7±9.9^{dB}	40.0±7.3^{dB}	43.3±8.0^{dA}
	0.03	76.7±8.0^{bcA}	63.3±14.^{bcB}	60.0±5.2^{bcA}	60.0±8.9^{bcA}
	0.06	76.7±6.1^{bcA}	66.7±6.7^{bB}	63.3±6.1^{bA}	63.3±6.2^{bA}
	0.12	83.3±6.1^{aB}	90.0±4.5^{aA}	73.3±8.4^{aB}	76.7±10.8^{aA}
S. polypodioides	0.0075	30.0±8.7^{eB}	36.7±3.3^{eA}	23.3±8.0^{eB}	36.7±8.0^{deA}
	0.015	60.0±5.2^{cdA}	43.3±6.1^{dB}	40.0±7.3^{dB}	43.3±6.1^{dA}
	0.03	63.3±9.5^{bcA}	60.0±7.3^{cB}	53.3±6.7^{bcB}	60.0±5.2^{bcA}
	0.06	66.7±2.3^{bB}	76.7±8.0^{bA}	56.7±3.3^{bB}	63.3±8.0^{abA}
	0.12	83.3±8.0^{aB}	86.7±5.3^{aA}	66.7±6.7^{aB}	70.0±6.8^{aA}
T. ornata	0.0075	40.0±5.2^{dB}	43.3±8.0^{dA}	30.0±8.6^{dB}	46.7±4.2^{dA}
	0.015	40.0±5.7^{dB}	50.0±12.4^{cdA}	43.3±8.0^{cB}	50.0±4.5^{cA}
	0.03	60.0±7.3^{cA}	53.3±6.7^{cB}	50.0±8.6^{bB}	53.3±6.7^{cA}
	0.06	76.7±6.1^{bA}	60.0±5.2^{bB}	50.0±10.0^{bB}	63.3±8.0^{bA}
	0.12	80.0±7.3^{aA}	83.3±6.1^{aA}	63.3±8.0^{aB}	70.0±10.0^{aA}
Gallic acid	0.0075	30.0±8.6^{e}		43.3±8.0^{de}	
	0.015	43.3±1.8^{cd}		46.7±6.7^{d}	
	0.03	53.3±6.7^{c}		53.3±6.7^{bc}	
	0.06	73.3±6.7^{b}		56.7±14.1^{b}	
	0.12	83.3±6.1^{a}		73.3±8.4^{a}	
Tannic acid	0.0075	26.7±8.4^{d}		40.0±8.9^{cd}	
	0.015	46.4±6.7^{c}		46.7±6.7^{bc}	
	0.03	56.7±6.1^{b}		53.3±4.2^{bc}	
	0.06	73.3±9.9^{a}		56.7±6.1^{b}	
	0.12	73.3±8.4^{a}		66.7±6.7^{a}	
Vijayneem	0.03	60.0±5.2^{a}		56.7±12.0^{a}	
Monocrotophos	0.03	80.0±5.2^{a}		60.0±8.9^{a}	

The same lowercase letters in a column for each plants and standards separately indicate no significant difference in the Tukey test ($P < 0.05$). The same uppercase letters indicate no significant difference in the Tukey test ($P < 0.05$) between F1 and F2 of oral and contact toxicity bioassays of adults and nymphs separately.

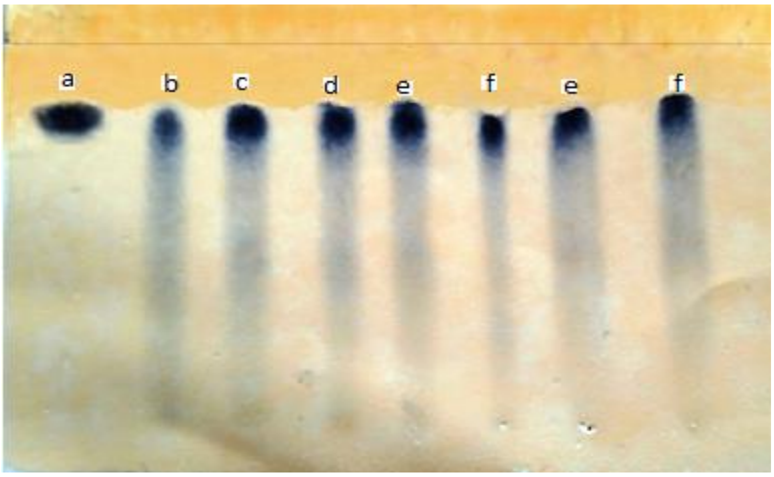

Figure S1. Photograph showing theTLC analysis of the standards (a)gallic acid, (b) tannic acid, (c)*S. wightii* F1, (d) *S. polypodioides*F1, (e)*T. ornata*F1, (f)*S. wightii* F2, (g) *S. polypodioides*F2 and (h) *T. ornata* F2. Tannic and gallic acids were processed in TLC 10 times, and samples were processed three times in TLC.

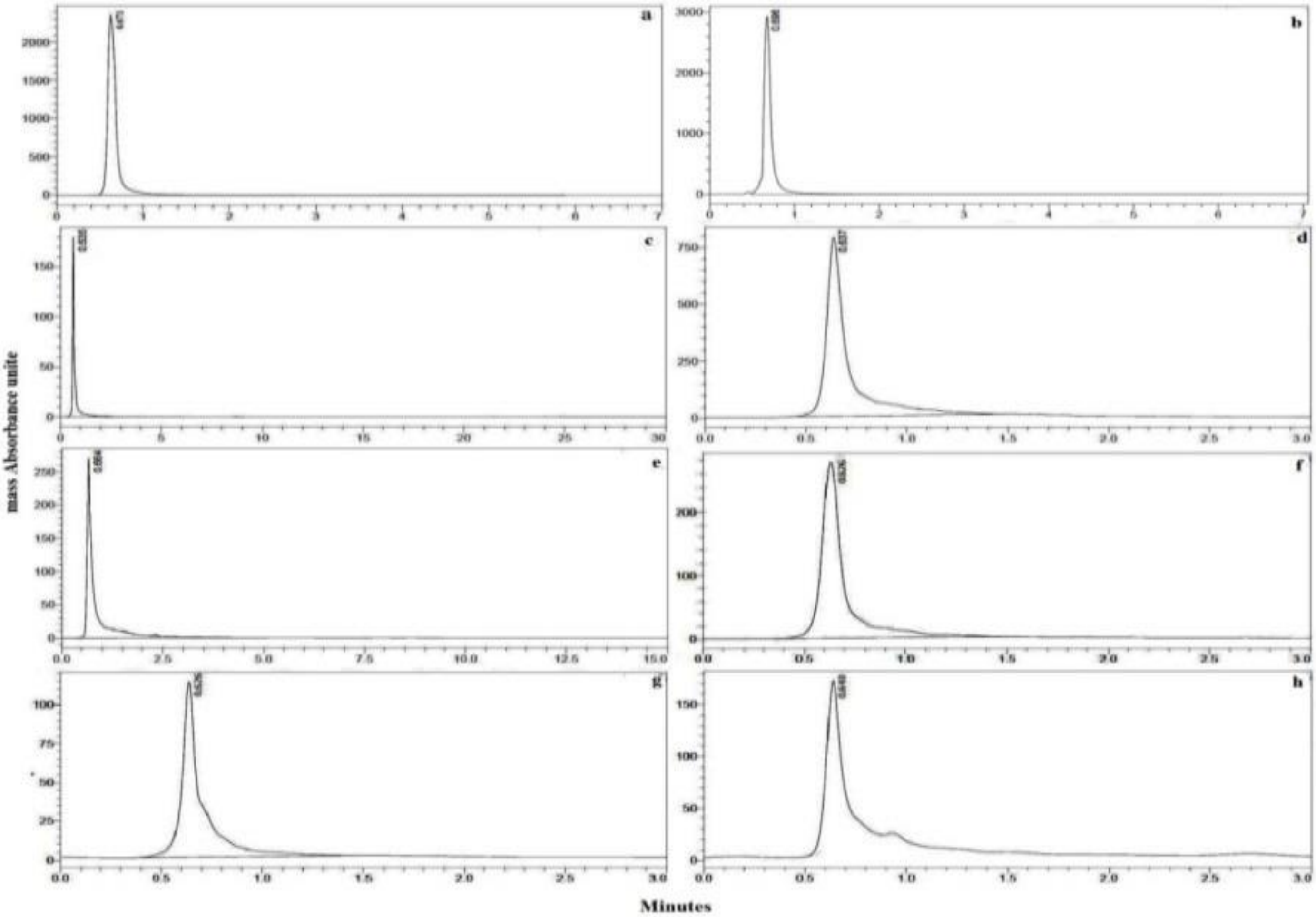

Figure S2. HPLC analysis of standard gallic acid (a) and tannic acid (b), and crude tanninsextracted from *S. wightii* (c),*S. polypodioides*(d) and *T. ornata* (e) using the Soxhlet method and *S. wightii* (f),*S. polypodioides*(g) and *T. ornata* (h) using the cold percolation method.

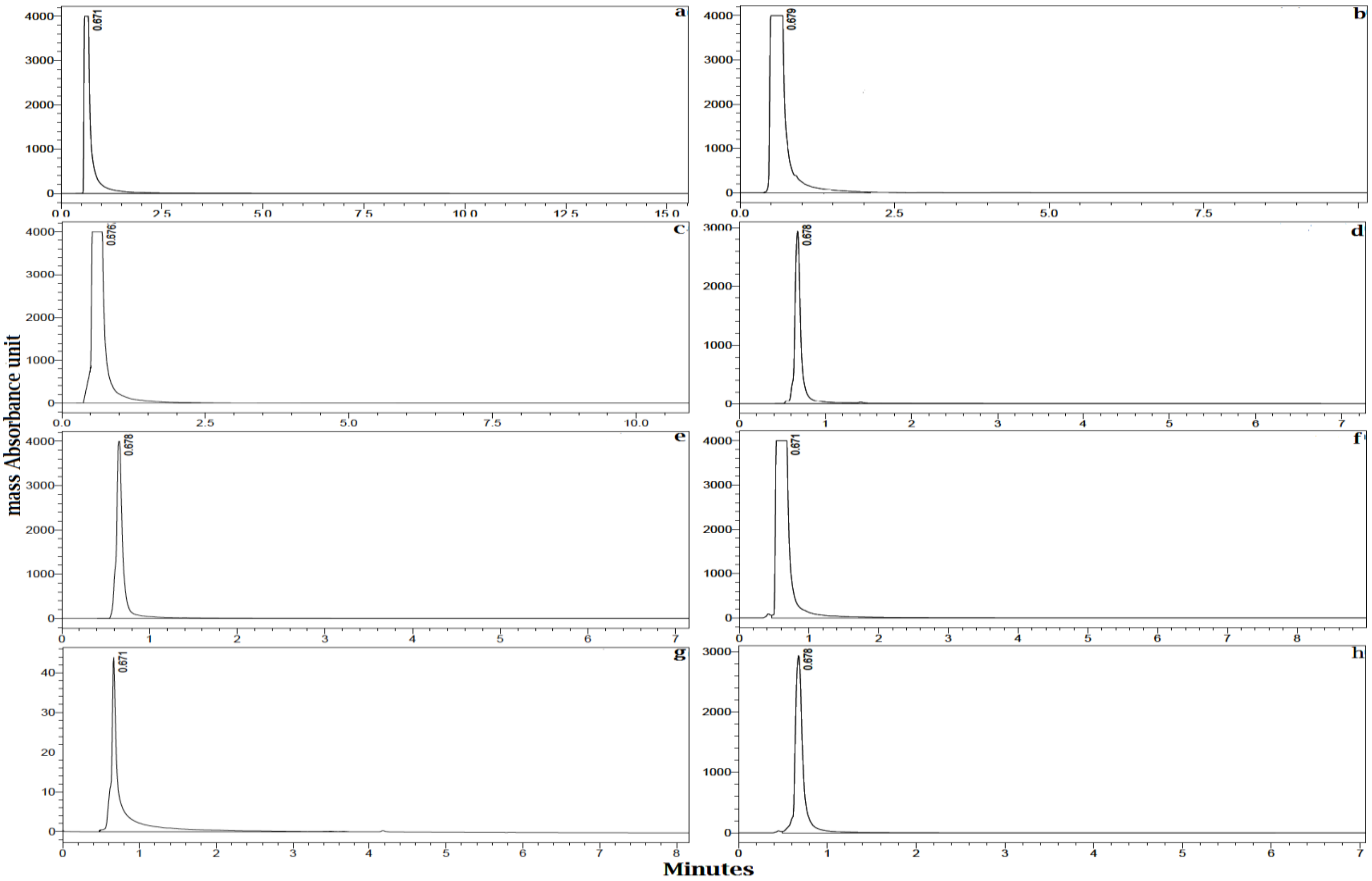

Figure S3. HPLC analysis of gallic acid (a) and tannic acid (b), andbrown algae *S. wightii* F1(c), *S. wightii* F2 (d), *S. polypodioides* F1 (e), *S. polypodioides* F2 (f), *T. ornata* F1 (g) and *T. ornata* F2 (h).

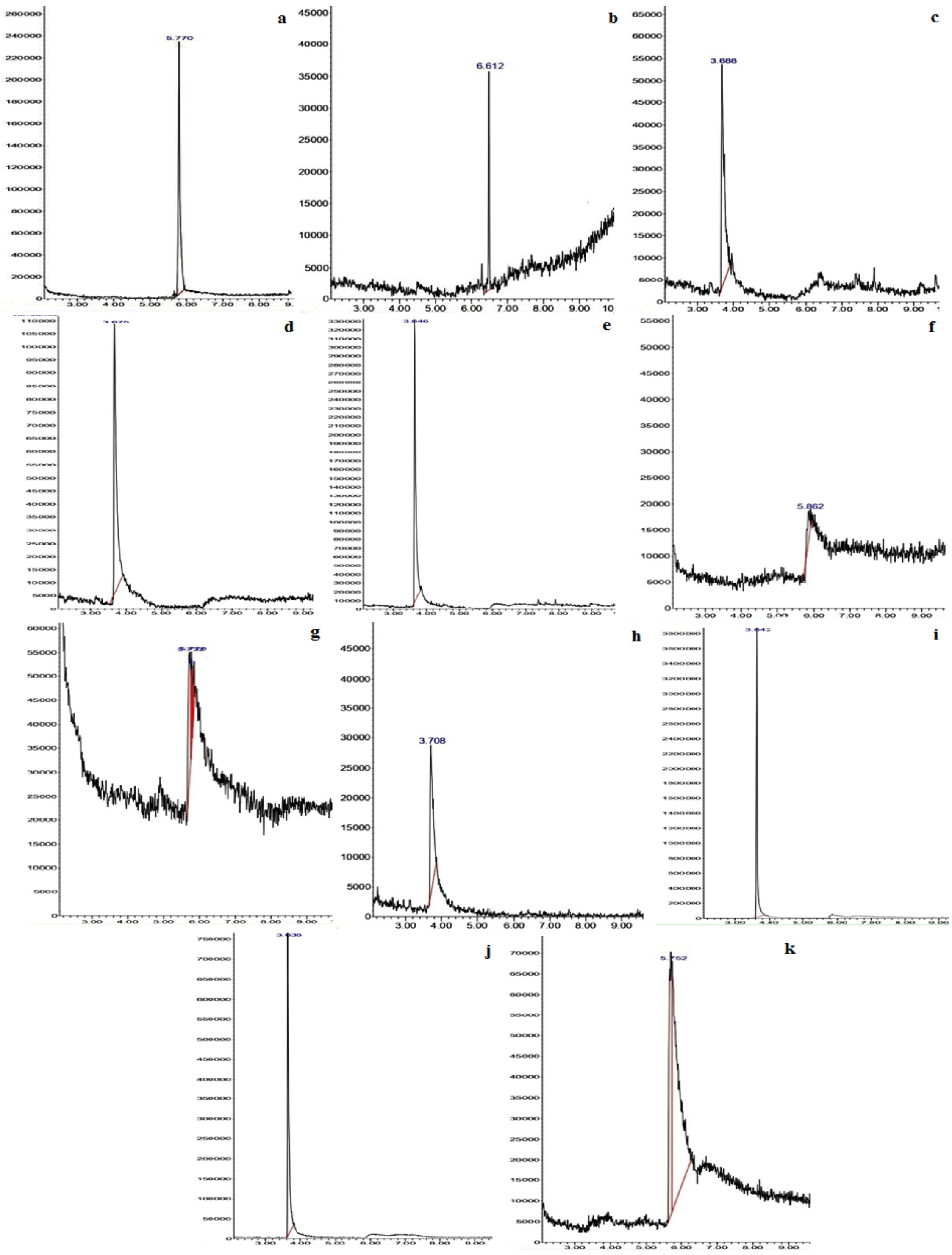

Figure S4. GC-MS analysis of gallic acid (a) and tannic acid (b), and drifted brown seaweed tannin crude extracts*S. wightii* (c), *S. polypodioides* (d) and *T. ornata* (e), *S. wightii* F1 (f), *S. polypodioides*F1 (g), *T. ornata*F1 (h), *S. wightii* F2 (i), *S. polypodioides*F2 (j) and *T. ornata* F2 (k).

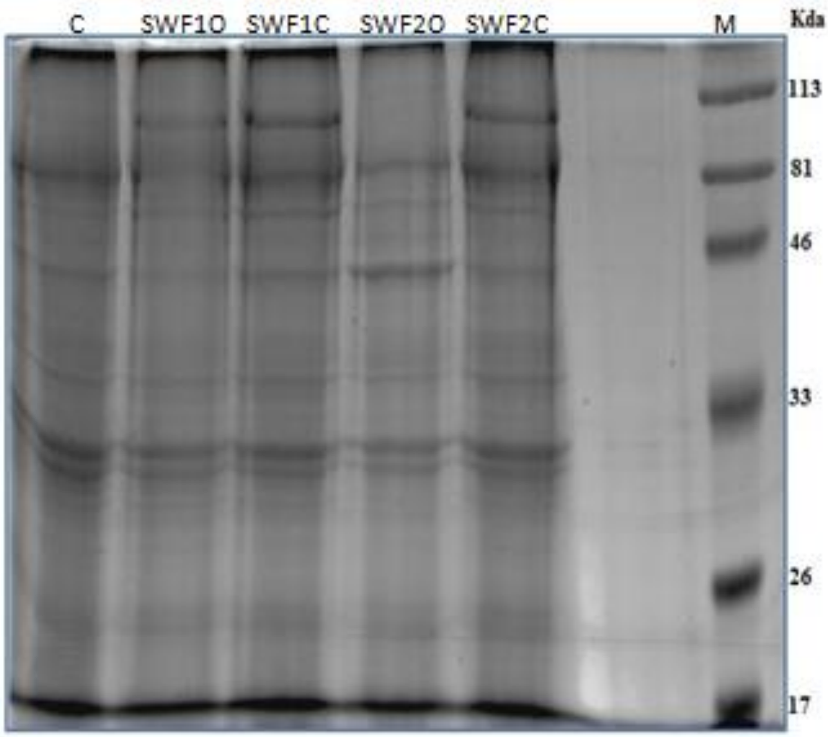

Figure S5. Effects of LC_{50} concentrations of seaweed tannin fractions on whole-body protein SDS-PAGE of *A. devastans* adults. C—control; SWF1O—*S.wightii* tannin F1 (oral toxicity); SWF2C—*S. wightii* tannin F1 (contact toxicity); SWF2O—*S. wightii* tannin F2 (oral toxicity); SWF2C— *S. wightii* tannin F2 (contact toxicity); and M—marker.
